# Supplementary material for: What’s not in the news headlines or titles of Alzheimer disease articles? #InMice
Source: PLoS Biol. 2021 Jun 15;19(6):e3001260. doi: 10.1371/journal.pbio.3001260 (PMC8205157; doi:10.1371/journal.pbio.3001260)
Supplement: S1 Table — (PDF) [file pbio.3001260.s001.pdf]

S1 Table: List of Declarative Papers (N=405)

Title	*PMID*
Elevated age-related cortical iron, ferritin and amyloid plaques in APP(swe)/PS1(deltaE9) transgenic mouse model of Alzheimer's disease	32118475
Gene delivery of a modified antibody to Aβ reduces progression of murine Alzheimer's disease	31887144
Mouse brain proteomics establishes MDGA1 and CACHD1 as in vivo substrates of the Alzheimer protease BACE1	31908000
Genetic perturbations of disease risk genes in mice capture transcriptomic signatures of late-onset Alzheimer's disease	31878951
Dampened Slow Oscillation Connectivity Anticipates Amyloid Deposition in the PS2APP Mouse Model of Alzheimer's Disease	31878336
Synergistic effects of APOE and sex on the gut microbiome of young EFAD transgenic mice	31861986
Circular RNA NF1-419 enhances autophagy to ameliorate senile dementia by binding Dynamin-1 and Adaptor protein 2 B1 in AD-like mice	31860870
Testing a MultiTEP-based combination vaccine to reduce Aβ and tau pathology in Tau22/5xFAD bigenic mice	31847886
Electroacupuncture attenuates cognition impairment via anti-neuroinflammation in an Alzheimer's disease animal model	31836020
EphA4 loss improves social memory performance and alters dendritic spine morphology without changes in amyloid pathology in a mouse model of Alzheimer's disease	31831046
MouseBytes, an open-access high-throughput pipeline and database for rodent touchscreen-based cognitive assessment	31825307
Tau- but not Aß -pathology enhances NMDAR-dependent depotentiation in AD-mouse models	31815648
C5aR agonist enhances phagocytosis of fibrillar and non-fibrillar Aβ amyloid and preserves memory in a mouse model of familial Alzheimer's disease	31809505
Brain targeting of 9c,11t-Conjugated Linoleic Acid, a natural calpain inhibitor, preserves memory and reduces Aβ and P25 accumulation in 5XFAD mice	31804596
Pharmacological inhibition of G9a/GLP restores cognition and reduces oxidative stress, neuroinflammation and β-Amyloid plaques in an early-onset Alzheimer's disease mouse model	31804189
Suppressing aberrant phospholipase D1 signaling in 3xTg Alzheimer's disease mouse model promotes synaptic resilience	31797996
Heterozygous CX3CR1 Deficiency in Microglia Restores Neuronal β-Amyloid Clearance Pathways and Slows Progression of Alzheimer's Like-Disease in PS1-APP Mice	31849963
High Dietary Iron Disrupts Iron Homeostasis and Induces Amyloid-β and Phospho-τ Expression in the Hippocampus of Adult Wild-Type and APP/PS1 Transgenic Mice	31373375
Epigenetic mechanisms underlying the effects of triptolide and tripchlorolide on the expression of neuroligin-1 in the hippocampus of APP/PS1 transgenic mice	31311385
A high-sucrose diet aggravates Alzheimer's disease pathology, attenuates hypothalamic leptin signaling, and impairs food-anticipatory activity in APPswe/PS1dE9 mice	31879131

Selective memory and behavioral alterations after ambient ultrafine particulate matter exposure in aged 3xTgAD Alzheimer's disease mice	31771615
Berberine mitigates cognitive decline in an Alzheimer's Disease Mouse Model by targeting both tau hyperphosphorylation and autophagic clearance	31810131
Optogenetic gamma stimulation rescues memory impairments in an Alzheimer's disease mouse model	31757962
Artemether Activation of AMPK/GSK3β(ser9)/Nrf2 Signaling Confers Neuroprotection towards β-Amyloid-Induced Neurotoxicity in 3xTg Alzheimer's Mouse Model	31871541
Chemerin-9 Peptide Enhances Memory and Ameliorates Aβ(1-42)-Induced Object Memory Impairment in Mice	31748466
Early Hippocampal Sharp-Wave Ripple Deficits Predict Later Learning and Memory Impairments in an Alzheimer's Disease Mouse Model	31747587
Studies on APP metabolism related to age-associated mitochondrial dysfunction in APP/PS1 transgenic mice	31744937
Plant sphingolipids promote extracellular vesicle release and alleviate amyloid-β pathologies in a mouse model of Alzheimer's disease	31727994
Retinal thinning of inner sub-layers is associated with cortical atrophy in a mouse model of Alzheimer's disease: a longitudinal multimodal in vivo study	31722748
Gut microbiota manipulation through probiotics oral administration restores glucose homeostasis in a mouse model of Alzheimer's disease	31813629
Polysaccharides from Lycium barbarum ameliorate amyloid pathology and cognitive functions in APP/PS1 transgenic mice	31715236
Tau deletion reduces plaque-associated BACE1 accumulation and decelerates plaque formation in a mouse model of Alzheimer's disease	31701556
ABCA7 haplodeficiency disturbs microglial immune responses in the mouse brain	31690660
N,N'-Diacetyl-p-phenylenediamine restores microglial phagocytosis and improves cognitive defects in Alzheimer's disease transgenic mice	31685616
Modified Huang-Lian-Jie-Du Decoction Ameliorates Aβ Synaptotoxicity in a Murine Model of Alzheimer's Disease	31781354
Annona atemoya leaf extract ameliorates cognitive impairment in amyloid-β injected Alzheimer's disease-like mouse model	31679404
Mega-Analysis of Gene Expression in Mouse Models of Alzheimer's Disease	31767574
Nicotinamide phosphoribosyltransferase-related signaling pathway in early Alzheimer's disease mouse models	31702813
Sphingosine Kinase 2 Potentiates Amyloid Deposition but Protects against Hippocampal Volume Loss and Demyelination in a Mouse Model of Alzheimer's Disease	31641049
Somatostatin Receptor Subtype-4 Regulates mRNA Expression of Amyloid-Beta Degrading Enzymes and Microglia Mediators of Phagocytosis in Brains of 3xTg-AD Mice	31630317
Strategy to enhance transgene expression in proximity of amyloid plaques in a mouse model of Alzheimer's disease	31754385
Long-Term Dabigatran Treatment Delays Alzheimer's Disease Pathogenesis in the TgCRND8 Mouse Model	31601371

Decreased levels of circulating trimethylamine N-oxide alleviate cognitive and pathological deterioration in transgenic mice: a potential therapeutic approach for Alzheimer's disease	31612864
Microglia drive APOE-dependent neurodegeneration in a tauopathy mouse model	31601677
Isoflurane mediated neuropathological and cognitive impairments in the triple transgenic Alzheimer's mouse model are associated with hippocampal synaptic deficits in an age-dependent manner	31600350
Bexarotene therapy ameliorates behavioral deficits and induces functional and molecular changes in very-old Triple Transgenic Mice model of Alzheimer´s disease	31596896
3D mapping reveals network-specific amyloid progression and subcortical susceptibility in mice	31602409
Shen-Zhi-Ling oral solution improves learning and memory ability in Alzheimer's disease mouse model	32186116
CSF1R inhibitor JNJ-40346527 attenuates microglial proliferation and neurodegeneration in P301S mice	31504240
Long-Term Pantethine Treatment Counteracts Pathologic Gene Dysregulation and Decreases Alzheimer's Disease Pathogenesis in a Transgenic Mouse Model	31267473
Mechanism of anti-dementia effects of mangiferin in a senescence accelerated mouse (SAMP8) model	31484797
Amyloid β-Induced Upregulation of Na(v)1.6 Underlies Neuronal Hyperactivity in Tg2576 Alzheimer's Disease Mouse Model	31537873
Norvaline Restores the BBB Integrity in a Mouse Model of Alzheimer's Disease	31540372
Genomic deletion of TLR2 induces aggravated white matter damage and deteriorated neurobehavioral functions in mouse models of Alzheimer's disease	31509519
The Role of Lysosomes in a Broad Disease-Modifying Approach Evaluated across Transgenic Mouse Models of Alzheimer's Disease and Parkinson's Disease and Models of Mild Cognitive Impairment	31505809
Network-guided analysis of hippocampal proteome identifies novel proteins that colocalize with Aβ in a mice model of early-stage Alzheimer's disease	31494281
Knockout of p75 neurotrophin receptor attenuates the hyperphosphorylation of Tau in pR5 mouse model	31479419
β-Carotene: A Natural Compound Improves Cognitive Impairment and Oxidative Stress in a Mouse Model of Streptozotocin-Induced Alzheimer's Disease	31480727
Group housing with young mice relieves Alzheimer's disease behaviors in aging mice	31599431
Neurite orientation dispersion and density imaging reveals white matter and hippocampal microstructure changes produced by Interleukin-6 in the TgCRND8 mouse model of amyloidosis	31472250
Dietary inulin alters the gut microbiome, enhances systemic metabolism and reduces neuroinflammation in an APOE4 mouse model	31461505
Enhancement of tripartite synapses as a potential therapeutic strategy for Alzheimer's disease: a preclinical study in rTg4510 mice	31439023
Space-like (56)Fe irradiation manifests mild, early sex-specific behavioral and neuropathological changes in wildtype and Alzheimer's-like transgenic mice	31431669
Potential synaptic plasticity-based Shenzhiling oral liquid for a SAD Mouse Model	31429527

Blockade of α4 integrins reduces leukocyte-endothelial interactions in cerebral vessels and improves memory in a mouse model of Alzheimer's disease	31427644
Visualizing Alzheimer's Disease Mouse Brain with Multispectral Optoacoustic Tomography using a Fluorescent probe, CDnir7	31427599
Serum and brain natural copper stable isotopes in a mouse model of Alzheimer's disease	31417103
Gestational Stress Augments Postpartum β-Amyloid Pathology and Cognitive Decline in a Mouse Model of Alzheimer's Disease	30561536
Icariin improves the cognitive function of APP/PS1 mice via suppressing endoplasmic reticulum stress	31400352
Glucocorticoid receptors modulate dendritic spine plasticity and microglia activity in an animal model of Alzheimer's disease	31394203
Fecal microbiota transplantation alleviated Alzheimer's disease-like pathogenesis in APP/PS1 transgenic mice	31383855
Twendee X Ameliorates Phosphorylated Tau, α-Synuclein and Neurovascular Dysfunction in Alzheimer's Disease Transgenic Mice With Chronic Cerebral Hypoperfusion	31383622
Late-stage Anle138b treatment ameliorates tau pathology and metabolic decline in a mouse model of human Alzheimer's disease tau	31370885
CNS cell type-specific gene profiling of P301S tau transgenic mice identifies genes dysregulated by progressive tau accumulation	31366728
p110δ PI3-Kinase Inhibition Perturbs APP and TNFα Trafficking, Reduces Plaque Burden, Dampens Neuroinflammation, and Prevents Cognitive Decline in an Alzheimer's Disease Mouse Model	31363064
Short amylin receptor antagonist peptides improve memory deficits in Alzheimer's disease mouse model	31358858
Acute restraint stress reverses impaired LTP in the hippocampal CA1 region in mouse models of Alzheimer's disease	31358853
Dual dose-dependent effects of fingolimod in a mouse model of Alzheimer's disease	31358793
Unilateral Focused Ultrasound-Induced Blood-Brain Barrier Opening Reduces Phosphorylated Tau from The rTg4510 Mouse Model	31410223
The Arctic/Swedish APP mutation alters the impact of chronic stress on cognition in mice	31231836
Malva parviflora extract ameliorates the deleterious effects of a high fat diet on the cognitive deficit in a mouse model of Alzheimer's disease by restoring microglial function via a PPAR-γ-dependent mechanism	31291963
(-)-Phenserine and the prevention of pre-programmed cell death and neuroinflammation in mild traumatic brain injury and Alzheimer's disease challenged mice	31295555
An one-pot two-step automated synthesis of [18F]T807 injection, its biodistribution in mice and monkeys, and a preliminary study in humans	31260447
A longitudinal multimodal in vivo molecular imaging study of the 3xTg-AD mouse model shows progressive early hippocampal and taurine loss	30816415
LC3-Associated Endocytosis Facilitates β-Amyloid Clearance and Mitigates Neurodegeneration in Murine Alzheimer's Disease	31257024
Plasma Pharmacokinetics of High-Affinity Transferrin Receptor Antibody-Erythropoietin Fusion Protein is a Function of Effector Attenuation in Mice	31199881

Oleocanthal-Rich Extra-Virgin Olive Oil Restores the Blood-Brain Barrier Function through NLRP3 Inflammasome Inhibition Simultaneously with Autophagy Induction in TgSwDI Mice	31244050
Red Ginseng Attenuates Aβ-Induced Mitochondrial Dysfunction and Aβ-mediated Pathology in an Animal Model of Alzheimer's Disease	31234321
Preventive Effect of Quercetin in a Triple Transgenic Alzheimer's Disease Mice Model	31226738
Adiponectin improves long-term potentiation in the 5XFAD mouse brain	31222110
Amelioration of Behavioral Impairments and Neuropathology by Antiepileptic Drug Topiramate in a Transgenic Alzheimer's Disease Model Mice, APP/PS1	31248209
Transcriptomic Insights into the Response of the Olfactory Bulb to Selenium Treatment in a Mouse Model of Alzheimer's Disease	31248178
Chronic Sleep Disruption Potentiates Locus Ceruleus Tauopathy in a Mouse Model of Alzheimer's Disease	31217341
Temporal progression of Alzheimer's disease in brains and intestines of transgenic mice	31284126
Early Electrophysiological Disintegration of Hippocampal Neural Networks in a Novel Locus Coeruleus Tau-Seeding Mouse Model of Alzheimer's Disease	31285742
"Dual Disease" TgAD/GSS mice exhibit enhanced Alzheimer's disease pathology and reveal PrP(C)-dependent secretion of Aβ	31189938
Ganglioside GQ1b ameliorates cognitive impairments in an Alzheimer's disease mouse model, and causes reduction of amyloid precursor protein	31186474
Long-term effects of maternal choline supplementation on CA1 pyramidal neuron gene expression in the Ts65Dn mouse model of Down syndrome and Alzheimer's disease	31180719
High Levels of β-Amyloid, Tau, and Phospho-Tau in Red Blood Cells as Biomarkers of Neuropathology in Senescence-Accelerated Mouse	31281579
Blast-Mediated Traumatic Brain Injury Exacerbates Retinal Damage and Amyloidosis in the APPswePSENd19e Mouse Model of Alzheimer's Disease	31247112
Enhancing face validity of mouse models of Alzheimer's disease with natural genetic variation	31150388
APOE genotype and sex affect microglial interactions with plaques in Alzheimer's disease mice	31113487
Cyclic O(3) exposure synergizes with aging leading to memory impairment in male APOE ε3, but not APOE ε4, targeted replacement mice	31207469
Comparing the effect of the novel ionic cocrystal of lithium salicylate proline (LISPRO) with lithium carbonate and lithium salicylate on memory and behavior in female APPswe/PS1dE9 Alzheimer's mice	31102295
Short-term fish oil supplementation applied in presymptomatic stage of Alzheimer's disease enhances microglial/macrophage barrier and prevents neuritic dystrophy in parietal cortex of 5xFAD mouse model	31095617
Rational design, cognition and neuropathology evaluation of QTC-4-MeOBnE in a streptozotocin-induced mouse model of sporadic Alzheimer's disease	31086208
Peripheral administration of human recombinant ApoJ/clusterin modulates brain beta-amyloid levels in APP23 mice	31077261
Aquaporin-4 reduces neuropathology in a mouse model of Alzheimer's disease by remodeling peri-plaque astrocyte structure	31068220

Long-term icariin treatment ameliorates cognitive deficits via CD4(+) T cell-mediated immuno-inflammatory responses in APP/PS1 mice	31190768
BIN1 recovers tauopathy-induced long-term memory deficits in mice and interacts with Tau through Thr(348) phosphorylation	31065832
High-throughput metabolomics and ingenuity pathway approach reveals the pharmacological effect and targets of Ginsenoside Rg1 in Alzheimer's disease mice	31065079
Estrogen-Dominant Ovarian Cycle Stages Are Associated with Neural Network Dysfunction and Cognitive and Behavioral Deficits in the hAPP-J20 Mouse Model of Alzheimer's Disease	31147386
APP-Mediated Signaling Prevents Memory Decline in Alzheimer's Disease Mouse Model	31042463
The db mutation improves memory in younger mice in a model of Alzheimer's disease	31034991
Clinical and Pathological Benefit of Twendee X in Alzheimer's Disease Transgenic Mice with Chronic Cerebral Hypoperfusion	31029568
Protein inhibitor of activated STAT1 Ser(503) phosphorylation-mediated Elk-1 SUMOylation promotes neuronal survival in APP/PS1 mice	30849179
SIRT3 mediates hippocampal synaptic adaptations to intermittent fasting and ameliorates deficits in APP mutant mice	31015456
Estrogen receptor α promotes Cav1.2 ubiquitination and degradation in neuronal cells and in APP/PS1 mice	31012223
Exposure to fluoride aggravates the impairment in learning and memory and neuropathological lesions in mice carrying the APP/PS1 double-transgenic mutation	31010414
LRP1 Has a Predominant Role in Production over Clearance of Aβ in a Mouse Model of Alzheimer's Disease	31004319
Nitazoxanide, an anti-parasitic drug, efficiently ameliorates learning and memory impairments in AD model mice	31000769
Beneficial effects of resveratrol and exercise training on cardiac and aortic function and structure in the 3xTg mouse model of Alzheimer's disease	31114160
Constipation in Tg2576 mice model for Alzheimer's disease associated with dysregulation of mechanism involving the mAChR signaling pathway and ER stress response	30978260
Differential effects of diet- and genetically-induced brain insulin resistance on amyloid pathology in a mouse model of Alzheimer's disease	30975165
Pharmacological activation of the nuclear receptor REV-ERB reverses cognitive deficits and reduces amyloid-β burden in a mouse model of Alzheimer's disease	30973894
Cognitive Decline, Cerebral-Spleen Tryptophan Metabolism, Oxidative Stress, Cytokine Production, and Regulation of the Txnip Gene in a Triple Transgenic Mouse Model of Alzheimer Disease	30980800
APOE genotype influences the gut microbiome structure and function in humans and mice: relevance for Alzheimer's disease pathophysiology	30958695
Activation of PPARA-mediated autophagy reduces Alzheimer disease-like pathology and cognitive decline in a murine model	30898012
Effect of genotype and age on cerebral [(18)F]FDG uptake varies between transgenic APP(Swe)-PS1(dE9) and Tg2576 mouse models of Alzheimer's disease	30952945
Female mice with apolipoprotein E4 domain interaction demonstrated impairments in spatial learning and memory performance and disruption of hippocampal cyto-architecture	30954674

Spatial Training Ameliorates Long-Term Alzheimer's Disease-Like Pathological Deficits by Reducing NLRP3 Inflammasomes in PR5 Mice	30560481
Multivariate MR biomarkers better predict cognitive dysfunction in mouse models of Alzheimer's disease	30940494
Impaired hippocampal-cortical coupling but preserved local synchrony during sleep in APP/PS1 mice modeling Alzheimer's disease	30926900
Functional networks are impaired by elevated tau-protein but reversible in a regulatable Alzheimer's disease mouse model	30917861
Prevention of Huntington's Disease-Like Behavioral Deficits in R6/1 Mouse by Tolfenamic Acid Is Associated with Decreases in Mutant Huntingtin and Oxidative Stress	31049134
The effects of omega-3 fatty acid deficiency during development on oxidative fatty acid degradation during maturity in a mouse model of Alzheimer's disease	31029017
Quantitative study of the capillaries within the white matter of the Tg2576 mouse model of Alzheimer's disease	30900389
Dietary Sargassum fusiforme improves memory and reduces amyloid plaque load in an Alzheimer's disease mouse model	30894635
Amyloid-β plaque formation and reactive gliosis are required for induction of cognitive deficits in App knock-in mouse models of Alzheimer's disease	30894120
Hippocampal Mossy Fibers Synapses in CA3 Pyramidal Cells Are Altered at an Early Stage in a Mouse Model of Alzheimer's Disease	30886015
Treatment with a brain-selective prodrug of 17β-estradiol improves cognitive function in Alzheimer's disease mice by regulating klf5-NF-κB pathway	30879099
Antibody-mediated clearance of tau in primary mouse microglial cultures requires Fcγ-receptor binding and functional lysosomes	30874605
Systemic LPS-induced Aβ-solubilization and clearance in AβPP-transgenic mice is diminished by heparanase overexpression	30872722
Synaptopodin Deficiency Ameliorates Symptoms in the 3xTg Mouse Model of Alzheimer's Disease	30872324
Urolithin A attenuates memory impairment and neuroinflammation in APP/PS1 mice	30871577
Recurrent herpes simplex virus-1 infection induces hallmarks of neurodegeneration and cognitive deficits in mice	30870531
Dual-drug loaded nanoparticles of Epigallocatechin-3-gallate (EGCG)/Ascorbic acid enhance therapeutic efficacy of EGCG in a APPswe/PS1dE9 Alzheimer's disease mice model	30876953
Deep convolutional neural networks for segmenting 3D in vivo multiphoton images of vasculature in Alzheimer disease mouse models	30865678
Comparative Gene-Expression Analysis of Alzheimer's Disease Progression with Aging in Transgenic Mouse Model	30862043
Role of GirK Channels in Long-Term Potentiation of Synaptic Inhibition in an In Vivo Mouse Model of Early Amyloid-β Pathology	30866445
Subacute to chronic Alzheimer-like alterations after controlled cortical impact in human tau transgenic mice	30846870
Administration of Repetitive Transcranial Magnetic Stimulation Attenuates Aβ (1-42)-Induced Alzheimer's Disease in Mice by Activating β-Catenin Signaling	30949496

Evaluating the applicability of mouse SINEs as an alternative normalization approach for RT-qPCR in brain tissue of the APP23 model for Alzheimer's disease	30849434
Traumatic Brain Injury by Weight-Drop Method Causes Transient Amyloid-β Deposition and Acute Cognitive Deficits in Mice	30944661
Delivery of BACE1 siRNA mediated by TARBP-BTP fusion protein reduces β-amyloid deposits in a transgenic mouse model of Alzheimer's disease	30837353
Anti-PrP(C) antibody rescues cognition and synapses in transgenic alzheimer mice	30911579
Emergence of synaptic and cognitive impairment in a mature-onset APP mouse model of Alzheimer's disease	30795807
Acute neuropathological consequences of short-term mechanical ventilation in wild-type and Alzheimer's disease mice	30795776
Trans ε viniferin decreases amyloid deposits and inflammation in a mouse transgenic Alzheimer model	30785960
Active immunization with norovirus P particle-based amyloid-β chimeric protein vaccine induces high titers of anti-Aβ antibodies in mice	30755174
Effects of Species-Specific Genetics on Alzheimer's Mouse Models	30731053
Activity-induced MEMRI cannot detect functional brain anomalies in the APPxPS1-Ki mouse model of Alzheimer's disease	30718666
Impaired Spatial Reorientation in the 3xTg-AD Mouse Model of Alzheimer's Disease	30718609
Exercise and resveratrol increase fracture resistance in the 3xTg-AD mouse model of Alzheimer's disease	30717730
Effects of manual acupuncture combined with donepezil in a mouse model of Alzheimer's disease	30843424
Conditional BDNF Delivery from Astrocytes Rescues Memory Deficits, Spine Density, and Synaptic Properties in the 5xFAD Mouse Model of Alzheimer Disease	30700530
PD-1/PD-L1 checkpoint blockade harnesses monocyte-derived macrophages to combat cognitive impairment in a tauopathy mouse model	30692527
Reduction of the expression of the late-onset Alzheimer's disease (AD) risk-factor BIN1 does not affect amyloid pathology in an AD mouse model	30692199
Repeated cold exposures protect a mouse model of Alzheimer's disease against cold-induced tau phosphorylation	30770297
Geniposide-mediated protection against amyloid deposition and behavioral impairment correlates with downregulation of mTOR signaling and enhanced autophagy in a mouse model of Alzheimer's disease	30684442
Heterochromatic genome instability and neurodegeneration sharing similarities with Alzheimer's disease in old Bmi1+/- mice	30679733
Qingxin kaiqiao fang ameliorates memory impairment and inhibits apoptosis in APP/PS1 double transgenic mice through the MAPK pathway	30774310
Reduced presynaptic vesicle stores mediate cellular and network plasticity defects in an early-stage mouse model of Alzheimer's disease	30670054
Genetic reduction of eEF2 kinase alleviates pathophysiology in Alzheimer's disease model mice	30667373
Expression Profiles of Long Noncoding RNAs in Intranasal LPS-Mediated Alzheimer's Disease Model in Mice	30809552

Activation of mGluR1 Mediates C1q-Dependent Microglial Phagocytosis of Glutamatergic Synapses in Alzheimer's Rodent Models	30652266
Early alterations in hippocampal perisomatic GABAergic synapses and network oscillations in a mouse model of Alzheimer's disease amyloidosis	30645585
Detection of Active Caspase-3 in Mouse Models of Stroke and Alzheimer's Disease with a Novel Dual Positron Emission Tomography/Fluorescent Tracer [(68)Ga]Ga-TC3-OGDOTA	30755766
Dissociable cognitive impairments in two strains of transgenic Alzheimer's disease mice revealed by a battery of object-based tests	30635592
Gene expression, proteome and calcium signaling alterations in immortalized hippocampal astrocytes from an Alzheimer's disease mouse model	30631041
Physical Activity Ameliorates Impaired Hippocampal Neurogenesis in the Tg4-42 Mouse Model of Alzheimer's Disease	31818124
Parvalbumin-Positive Neuron Loss and Amyloid-β Deposits in the Frontal Cortex of Alzheimer's Disease-Related Mice	31743995
Granisetron Alleviates Alzheimer's Disease Pathology in TgSwDI Mice Through Calmodulin-Dependent Protein Kinase II/cAMP-Response Element Binding Protein Pathway	31683487
MK0677, a Ghrelin Mimetic, Improves Neurogenesis but Fails to Prevent Hippocampal Lesions in a Mouse Model of Alzheimer's Disease Pathology	31594237
The Additive Effects of Low Dose Intake of Ferulic Acid, Phosphatidylserine and Curcumin, Not Alone, Improve Cognitive Function in APPswe/PS1dE9 Transgenic Mice	31582657
Global Metabolic Shifts in Age and Alzheimer's Disease Mouse Brains Pivot at NAD+/NADH Redox Sites	31356210
High Contrast and Resolution Labeling of Amyloid Plaques in Tissue Sections from APP-PS1 Mice and Humans with Alzheimer's Disease with the Zinc Chelator HQ-O: Practical and Theoretical Considerations	31345150
5xFAD Mice Display Sex-Dependent Inflammatory Gene Induction During the Prodromal Stage of Alzheimer's Disease	31322556
Vibrating Tail, Digging, Body/Face Interaction, and Lack of Barbering: Sex-Dependent Behavioral Signatures of Social Dysfunction in 3xTg-AD Mice as Compared to Mice with Normal Aging	31156176
Acute Anti-Inflammatory Markers ITIH4 and AHSG in Mice Brain of a Novel Alzheimer's Disease Model	30958362
Effect of Chinese herbal compound GAPT on the early brain glucose metabolism of APP/PS1 transgenic mice	30957587
Reducing ADAMTS-3 Inhibits Amyloid β Deposition in App Knock-in Mouse	30828067
Changes in Chemokines and Chemokine Receptors Expression in a Mouse Model of Alzheimer's Disease	30745834
MMP13 inhibition rescues cognitive decline in Alzheimer transgenic mice via BACE1 regulation	30596903
Tocotrienol Rich Fraction Supplementation Modulate Brain Hippocampal Gene Expression in APPswe/PS1dE9 Alzheimer's Disease Mouse Model	30507571
Diet-induced insulin resistance elevates hippocampal glutamate as well as VGLUT1 and GFAP expression in AβPP/PS1 mice	30472734

Olive Biophenols Reduces Alzheimer's Pathology in SH-SY5Y Cells and APPswe Mice	30598025
Harnessing Genetic Complexity to Enhance Translatability of Alzheimer's Disease Mouse Models: A Path toward Precision Medicine	30595332
Pridopidine stabilizes mushroom spines in mouse models of Alzheimer's disease by acting on the sigma-1 receptor	30594810
Low Phytanic Acid-Concentrated DHA Prevents Cognitive Deficit and Regulates Alzheimer Disease Mediators in an ApoE(-/-) Mice Experimental Model	30577526
Cinnamic acid activates PPARα to stimulate Lysosomal biogenesis and lower Amyloid plaque pathology in an Alzheimer's disease mouse model	30578827
Investigation of the neuroprotective effects of crocin via antioxidant activities in HT22 cells and in mice with Alzheimer's disease	30569175
Combined treatment with the phenolics (-)-epigallocatechin-3-gallate and ferulic acid improves cognition and reduces Alzheimer-like pathology in mice	30563837
Association of gut microbiota composition and function with a senescence-accelerated mouse model of Alzheimer's Disease using 16S rRNA gene and metagenomic sequencing analysis	30562162
Genome-wide RNAseq study of the molecular mechanisms underlying microglia activation in response to pathological tau perturbation in the rTg4510 tau transgenic animal model	30558641
Ovarian Cycle Stages Modulate Alzheimer-Related Cognitive and Brain Network Alterations in Female Mice	30627643
Mitochondrial biogenesis mediated by melatonin in an APPswe/PS1dE9 transgenic mice model	30303857
Diffusion MRI detects longitudinal white matter changes in the 3xTg-AD mouse model of Alzheimer's disease	30543850
5-HIAA induces neprilysin to ameliorate pathophysiology and symptoms in a mouse model for Alzheimer's disease	30537985
Chronic Intermittent Hypoxia Induces Robust Astrogliosis in an Alzheimer's Disease-Relevant Mouse Model	30529693
Neuroprotection and improvement of the histopathological and behavioral impairments in a murine Alzheimer's model treated with Zephyranthes carinata alkaloids	30530228
Nurr1 (NR4A2) regulates Alzheimer's disease-related pathogenesis and cognitive function in the 5XFAD mouse model	30515963
APOE genotype affects metabolic and Alzheimer-related outcomes induced by Western diet in female EFAD mice	30509127
Effect of Cuzhi liquid on learning and memory dysfunction in a mouse model of Alzheimer's disease	32186136
Lipocalin 2 contributes to brain iron dysregulation but does not affect cognition, plaque load, and glial activation in the J20 Alzheimer mouse model	30501637
Temporal and regional progression of Alzheimer's disease-like pathology in 3xTg-AD mice	30488653
Alzheimer's Disease Phenotype or Inflammatory Insult Does Not Alter Function of L-Type Amino Acid Transporter 1 in Mouse Blood-Brain Barrier and Primary Astrocytes	30488131
Defined astrocytic expression of human amyloid precursor protein in Tg2576 mouse brain	30485540
Selective reduction of APP-BACE1 activity improves memory via NMDA-NR2B receptor-mediated mechanisms in aged PDAPP mice	30572184

The Anti-amyloid Compound DO1 Decreases Plaque Pathology and Neuroinflammation-Related Expression Changes in 5xFAD Transgenic Mice	30472115
Contributions of a high-fat diet to Alzheimer's disease-related decline: A longitudinal behavioural and structural neuroimaging study in mouse models	30503215
Intranasal insulin activates Akt2 signaling pathway in the hippocampus of wild-type but not in APP/PS1 Alzheimer model mice	30554086
Chronic low dose of AM404 ameliorates the cognitive impairment and pathological features in hyperglycemic 3xTg-AD mice	30426182
Curcumin Ameliorates Memory Deficits by Enhancing Lactate Content and MCT2 Expression in APP/PS1 Transgenic Mouse Model of Alzheimer's Disease	30312017
Administration of the benzodiazepine midazolam increases tau phosphorylation in the mouse brain	30508732
Distinct disruptions in Land's cycle remodeling of glycerophosphocholines in murine cortex mark symptomatic onset and progression in two Alzheimer's disease mouse models	30040874
Deceleration of the neurodegenerative phenotype in pyroglutamate-Aβ accumulating transgenic mice by oral treatment with the Aβ oligomer eliminating compound RD2	30391539
Restoring microglial and astroglial homeostasis using DNA immunization in a Down Syndrome mouse model	30389461
MicroRNA-98 reduces amyloid β-protein production and improves oxidative stress and mitochondrial dysfunction through the Notch signaling pathway via HEY2 in Alzheimer's disease mice	30365070
A Novel Peroxidase Mimics and Ameliorates Alzheimer's Disease-Related Pathology and Cognitive Decline in Mice	30352982
Noggin rescues age-related stem cell loss in the brain of senescent mice with neurodegenerative pathology	30352848
NMDA receptors mediate synaptic depression, but not spine loss in the dentate gyrus of adult amyloid Beta (Aβ) overexpressing mice	30352630
Platelets isolated from an Alzheimer mouse damage healthy cortical vessels and cause inflammation in an organotypic ex vivo brain slice model	30341392
Modulation of astrocyte reactivity improves functional deficits in mouse models of Alzheimer's disease	30322407
ER stress is not elevated in the 5XFAD mouse model of Alzheimer's disease	30315100
The small molecule CA140 inhibits the neuroinflammatory response in wild-type mice and a mouse model of AD	30309372
Human neural stem cell transplantation improves cognition in a murine model of Alzheimer's disease	30283042
Integrative approach to sporadic Alzheimer's disease: deficiency of TYROBP in cerebral Aβ amyloidosis mouse normalizes clinical phenotype and complement subnetwork molecular pathology without reducing Aβ burden	30283032
Integrative approach to sporadic Alzheimer's disease: deficiency of TYROBP in a tauopathy mouse model reduces C1q and normalizes clinical phenotype while increasing spread and state of phosphorylation of tau	30283031

Chronic oral application of a periodontal pathogen results in brain inflammation, neurodegeneration and amyloid beta production in wild type mice	30281647
Chronic noise exposure exacerbates AD-like neuropathology in SAMP8 mice in relation to Wnt signaling in the PFC and hippocampus	30279527
L-Norvaline Reverses Cognitive Decline and Synaptic Loss in a Murine Model of Alzheimer's Disease	30288668
[(18)F]F-DPA for the detection of activated microglia in a mouse model of Alzheimer's disease	30317069
Caspase-1 inhibition alleviates cognitive impairment and neuropathology in an Alzheimer's disease mouse model	30254377
Blood-derived plasminogen drives brain inflammation and plaque deposition in a mouse model of Alzheimer's disease	30254165
Alzheimer's associated amyloid and tau deposition co-localizes with a homeostatic myelin repair pathway in two mouse models of post-stroke mixed dementia	30249297
Centella asiatica attenuates hippocampal mitochondrial dysfunction and improves memory and executive function in β-amyloid overexpressing mice	30253196
Sirt1 enhances tau exon 10 inclusion and improves spatial memory of Htau mice	30243024
Microglia prevent peripheral immune cell invasion and promote an anti-inflammatory environment in the brain of APP-PS1 transgenic mice	30241479
Time course of focused ultrasound effects on β-amyloid plaque pathology in the TgCRND8 mouse model of Alzheimer's disease	30232364
Efficacy of chronic BACE1 inhibition in PS2APP mice depends on the regional Aβ deposition rate and plaque burden at treatment initiation	30429879
Blood-brain barrier integrity in a mouse model of Alzheimer's disease with or without acute 3D6 immunotherapy	30201212
Combined adult neurogenesis and BDNF mimic exercise effects on cognition in an Alzheimer's mouse model	30190379
Short-Term Fish Oil Treatment Changes the Composition of Phospholipids While Not Affecting the Expression of Mfsd2a Omega-3 Transporter in the Brain and Liver of the 5xFAD Mouse Model of Alzheimer's Disease	30200627
Human Alzheimer's disease gene expression signatures and immune profile in APP mouse models: a discrete transcriptomic view of Aβ plaque pathology	30189875
Molecular interplay between hyperactive mammalian target of rapamycin signaling and Alzheimer's disease neuropathology in the NS-Pten knockout mouse model	29965873
Isoform-specific hyperactivation of calpain-2 occurs presymptomatically at the synapse in Alzheimer's disease mice and correlates with memory deficits in human subjects	30177812
Hydroxyurea attenuates oxidative, metabolic, and excitotoxic stress in rat hippocampal neurons and improves spatial memory in a mouse model of Alzheimer's disease	30245242
Altered Expression of Small Intestinal Drug Transporters and Hepatic Metabolic Enzymes in a Mouse Model of Familial Alzheimer's Disease	30074800
Paradoxical effects of mutant ubiquitin on Aβ plaque formation in an Alzheimer mouse model	30216939
Riluzole reduces amyloid beta pathology, improves memory, and restores gene expression changes in a transgenic mouse model of early-onset Alzheimer's disease	30108205

Novel Quantitative Analyses of Spontaneous Synaptic Events in Cortical Pyramidal Cells Reveal Subtle Parvalbumin-Expressing Interneuron Dysfunction in a Knock-In Mouse Model of Alzheimer's Disease	30105300
Micro-RNA-137 Inhibits Tau Hyperphosphorylation in Alzheimer's Disease and Targets the CACNA1C Gene in Transgenic Mice and Human Neuroblastoma SH-SY5Y Cells	30102687
Sex Differences in Neuropathology and Cognitive Behavior in APP/PS1/tau Triple-Transgenic Mouse Model of Alzheimer's Disease	30099679
Rapid and reversible impairment of episodic memory by a high-fat diet in mice	30097632
Augmented Insulin and Leptin Resistance of High Fat Diet-Fed APPswe/PS1dE9 Transgenic Mice Exacerbate Obesity and Glycemic Dysregulation	30096853
Altered glutamate clearance in ascorbate deficient mice increases seizure susceptibility and contributes to cognitive impairment in APP/PSEN1 mice	30172223
Genetic deletion of CB(1) cannabinoid receptors exacerbates the Alzheimer-like symptoms in a transgenic animal model	30096288
High-fat diet protects the blood-brain barrier in an Alzheimer's disease mouse model	30079520
The Protective Effects of Clams on Hypercholesterolemia in Late-Stage Triple-Transgenic Alzheimer's Diseased Mice Hearts	30071640
Cognitive and emotional alterations in App knock-in mouse models of Aβ amyloidosis	30055565
Assessment of brain beta-amyloid deposition in transgenic mouse models of Alzheimer's disease with PET imaging agents (18)F-flutemetamol and (18)F-florbetaben	30053803
Oxidation of KCNB1 channels in the human brain and in mouse model of Alzheimer's disease	30050035
Early Activation of Astrocytes does not Affect Amyloid Plaque Load in an Animal Model of Alzheimer's Disease	30032411
In vivo tracking of intravenously injected mesenchymal stem cells in an Alzheimer's animal model	30008224
Aβ Oligomer Elimination Restores Cognition in Transgenic Alzheimer's Mice with Full-blown Pathology	30003517
Concurrent cell type-specific isolation and profiling of mouse brains in inflammation and Alzheimer's disease	29997299
Deficiency in the transcription factor NRF2 worsens inflammatory parameters in a mouse model with combined tauopathy and amyloidopathy	30029164
Bidirectional modulation of Alzheimer phenotype by alpha-synuclein in mice and primary neurons	29995210
Ligustilide Ameliorates Memory Deficiency in APP/PS1 Transgenic Mice via Restoring Mitochondrial Dysfunction	30079347
The effects of treadmill exercise on autophagy in hippocampus of APP/PS1 transgenic mice	29672446
Quasi-Periodic Patterns of Neural Activity improve Classification of Alzheimer's Disease in Mice	29968786
Aspirin Induces Lysosomal Biogenesis and Attenuates Amyloid Plaque Pathology in a Mouse Model of Alzheimer's Disease via PPARα	29967008
Whole brain vascular imaging in a mouse model of Alzheimer's disease with two-photon microscopy	29998647

Impact of Neurodegenerative Diseases on Drug Binding to Brain Tissues: From Animal Models to Human Samples	29675823
Quantitative proteomics of acutely-isolated mouse microglia identifies novel immune Alzheimer's disease-related proteins	29954413
Spontaneous development of Alzheimer's disease-associated brain pathology in a Shugoshin-1 mouse cohesinopathy model	29943428
New role of P2X7 receptor in an Alzheimer's disease mouse model	29934546
Hepatitis B core VLP-based mis-disordered tau vaccine elicits strong immune response and alleviates cognitive deficits and neuropathology progression in Tau.P301S mouse model of Alzheimer's disease and frontotemporal dementia	29914543
TSPO ligand PK11195 improves Alzheimer-related outcomes in aged female 3xTg-AD mice	29925037
Nrf2 Deficiency Exacerbates Obesity-Induced Oxidative Stress, Neurovascular Dysfunction, Blood-Brain Barrier Disruption, Neuroinflammation, Amyloidogenic Gene Expression, and Cognitive Decline in Mice, Mimicking the Aging Phenotype	29905772
An isoform-selective p38α mitogen-activated protein kinase inhibitor rescues early entorhinal cortex dysfunctions in a mouse model of Alzheimer's disease	30007168
Intradermal active full-length DNA Aβ42 immunization via electroporation leads to high anti-Aβ antibody levels in wild-type mice	29958693
An optimized method for enrichment of whole brain-derived extracellular vesicles reveals insight into neurodegenerative processes in a mouse model of Alzheimer's disease	29894726
Impaired AMPA signaling and cytoskeletal alterations induce early synaptic dysfunction in a mouse model of Alzheimer's disease	29877034
Heme and hemoglobin suppress amyloid β-mediated inflammatory activation of mouse astrocytes	29871926
Dihydromyricetin inhibits microglial activation and neuroinflammation by suppressing NLRP3 inflammasome activation in APP/PS1 transgenic mice	29869390
Evaluation of neuropathological effects of a high-fat high-sucrose diet in middle-aged male C57BL6/J mice	29890051
Oral administration of Pantoea agglomerans-derived lipopolysaccharide prevents metabolic dysfunction and Alzheimer's disease-related memory loss in senescence-accelerated prone 8 (SAMP8) mice fed a high-fat diet	29856882
Environmental enrichment reverses Aβ pathology during pregnancy in a mouse model of Alzheimer's disease	29855361
Antibody-Based In Vivo PET Imaging Detects Amyloid-β Reduction in Alzheimer Transgenic Mice After BACE-1 Inhibition	29853653
Huatuo Zaizao pill ameliorates cognitive impairment of APP/PS1 transgenic mice by improving synaptic plasticity and reducing Aβ deposition	29843688
Altered plasma arginine metabolome precedes behavioural and brain arginine metabolomic profile changes in the APPswe/PS1ΔE9 mouse model of Alzheimer's disease	29802260
Efficient clearance of Aβ protofibrils in AβPP-transgenic mice treated with a brain-penetrating bifunctional antibody	29793530
Cannabinoid CB(2) receptors in the mouse brain: relevance for Alzheimer's disease	29793509

Pathology of nNOS-Expressing GABAergic Neurons in Mouse Model of Alzheimer's Disease	29782905
[(18)F]FMPEP-d(2) PET imaging shows age- and genotype-dependent impairments in the availability of cannabinoid receptor 1 in a mouse model of Alzheimer's disease	29909177
AMPA-ergic regulation of amyloid-β levels in an Alzheimer's disease mouse model	29764453
Ameliorative potential of desalted Salicornia europaea L. extract in multifaceted Alzheimer's-like scopolamine-induced amnesic mice model	29740000
URMC-099 facilitates amyloid-β clearance in a murine model of Alzheimer's disease	29729668
Elevated levels of brain homocysteine directly modulate the pathological phenotype of a mouse model of tauopathy	29728702
Generation of App knock-in mice reveals deletion mutations protective against Alzheimer's disease-like pathology	29728560
C/EBPβ regulates delta-secretase expression and mediates pathogenesis in mouse models of Alzheimer's disease	29725016
Histone acetylation maps in aged mice developmentally exposed to lead: epigenetic drift and Alzheimer-related genes	29722544
Development of GMP-1 a molecular chaperone network modulator protecting mitochondrial function and its assessment in fly and mice models of Alzheimer's disease	29704317
Repeat propofol anesthesia does not exacerbate plaque deposition or synapse loss in APP/PS1 Alzheimer's disease mice	29699479
Maternal imprinting on cognition markers of wild type and transgenic Alzheimer's disease model mice	29691440
Reversal of memory and neuropsychiatric symptoms and reduced tau pathology by selenium in 3xTg-AD mice	29691439
Lack of human-like extracellular sortilin neuropathology in transgenic Alzheimer's disease model mice and macaques	29690919
Modulatory Effects of Fingolimod (FTY720) on the Expression of Sphingolipid Metabolism-Related Genes in an Animal Model of Alzheimer's Disease	29687345
Age-Related Increase of Insulin-Degrading Enzyme Is Inversely Correlated with Cognitive Function in APPswe/PS1dE9 Mice	29680859
Spatial reversal learning defect coincides with hypersynchronous telencephalic BOLD functional connectivity in APP(NL-F/NL-F) knock-in mice	29674739
Immunohistochemical Evidence from APP-Transgenic Mice for Glutaminyl Cyclase as Drug Target to Diminish pE-Abeta Formation	29673150
Hippocampal mutant APP and amyloid beta-induced cognitive decline, dendritic spine loss, defective autophagy, mitophagy and mitochondrial abnormalities in a mouse model of Alzheimer's disease	29408999
Evaluation of Animal Models by Comparison with Human Late-Onset Alzheimer's Disease	29656362
Scutellarin Mitigates Aβ-Induced Neurotoxicity and Improves Behavior Impairments in AD Mice	29642616
Autophagy is increased following either pharmacological or genetic silencing of mGluR5 signaling in Alzheimer's disease mouse models	29631635
Combination anti-Aβ treatment maximizes cognitive recovery and rebalances mTOR signaling in APP mice	29626114

Genetically reducing mTOR signaling rescues central insulin dysregulation in a mouse model of Alzheimer's disease	29729422
The diabetes drug liraglutide reverses cognitive impairment in mice and attenuates insulin receptor and synaptic pathology in a non-human primate model of Alzheimer's disease	29435980
TREM2-activating antibodies abrogate the negative pleiotropic effects of the Alzheimer's disease variant Trem2(R47H) on murine myeloid cell function	29599291
Dynamic SAP102 expression in the hippocampal subregions of rats and APP/PS1 mice of various ages	29574717
Intrahippocampal injection of a lentiviral vector expressing neurogranin enhances cognitive function in 5XFAD mice	29568074
Granulocyte-macrophage colony-stimulating factor neuroprotective activities in Alzheimer's disease mice	29573847
Nav1.1-Overexpressing Interneuron Transplants Restore Brain Rhythms and Cognition in a Mouse Model of Alzheimer's Disease	29551491
RAGE mediates Aβ accumulation in a mouse model of Alzheimer's disease via modulation of β- and γ-secretase activity	29329433
T(1), diffusion tensor, and quantitative magnetization transfer imaging of the hippocampus in an Alzheimer's disease mouse model	29545212
Behavioral and SCN neurophysiological disruption in the Tg-SwDI mouse model of Alzheimer's disease	29540298
Deuterated polyunsaturated fatty acids reduce brain lipid peroxidation and hippocampal amyloid β-peptide levels, without discernable behavioral effects in an APP/PS1 mutant transgenic mouse model of Alzheimer's disease	29579687
Protective effects of 7,8-dihydroxyflavone on neuropathological and neurochemical changes in a mouse model of Alzheimer's disease	29510124
Fisetin Reduces the Impact of Aging on Behavior and Physiology in the Rapidly Aging SAMP8 Mouse	28575152
Human Umbilical Cord Blood Serum-derived α-Secretase: Functional Testing in Alzheimer's Disease Mouse Models	29560732
Early long-term administration of the CSF1R inhibitor PLX3397 ablates microglia and reduces accumulation of intraneuronal amyloid, neuritic plaque deposition and pre-fibrillar oligomers in 5XFAD mouse model of Alzheimer's disease	29490706
SLAB51 Probiotic Formulation Activates SIRT1 Pathway Promoting Antioxidant and Neuroprotective Effects in an AD Mouse Model	29492848
Muscarinic Receptor-Dependent Long Term Depression in the Perirhinal Cortex and Recognition Memory are Impaired in the rTg4510 Mouse Model of Tauopathy	29484523
Toxoplasma gondii alters NMDAR signaling and induces signs of Alzheimer's disease in wild-type, C57BL/6 mice	29471842
Solid lipid curcumin particles provide greater anti-amyloid, anti-inflammatory and neuroprotective effects than curcumin in the 5xFAD mouse model of Alzheimer's disease	29471781
The Cyanthin Diterpenoid and Sesterterpene Constituents of Hericium erinaceus Mycelium Ameliorate Alzheimer's Disease-Related Pathologies in APP/PS1 Transgenic Mice	29463001

Nuclear receptor agonist-driven modification of inflammation and amyloid pathology enhances and sustains cognitive improvements in a mouse model of Alzheimer's disease	29448961
Comprehensive analysis of differentially expressed profiles of Alzheimer's disease associated circular RNAs in an Alzheimer's disease mouse model	29448241
MH84 improves mitochondrial dysfunction in a mouse model of early Alzheimer's disease	29433569
CA1 pyramidal neuron gene expression mosaics in the Ts65Dn murine model of Down syndrome and Alzheimer's disease following maternal choline supplementation	29394516
LINGO-1 antibody ameliorates myelin impairment and spatial memory deficits in the early stage of 5XFAD mice	29427384
Reduced cooperativity of voltage-gated sodium channels in the hippocampal interneurons of an aged mouse model of Alzheimer's disease	29427204
NAD(+) supplementation normalizes key Alzheimer's features and DNA damage responses in a new AD mouse model with introduced DNA repair deficiency	29432159
Tau passive immunization blocks seeding and spread of Alzheimer hyperphosphorylated Tau-induced pathology in 3 × Tg-AD mice	29386065
Ultramicronized palmitoylethanolamide rescues learning and memory impairments in a triple transgenic mouse model of Alzheimer's disease by exerting anti-inflammatory and neuroprotective effects	29382825
Novel botanical drug DA-9803 prevents deficits in Alzheimer's mouse models	29378621
Wnt signaling loss accelerates the appearance of neuropathological hallmarks of Alzheimer's disease in J20-APP transgenic and wild-type mice	29240990
Innate immune alterations are elicited in microglial cells before plaque deposition in the Alzheimer's disease mouse model 5xFAD	29367720
Intracellular Ca(2+) stores control in vivo neuronal hyperactivity in a mouse model of Alzheimer's disease	29358403
Dietary Total Prenylflavonoids from the Fruits of Psoralea corylifolia L. Prevents Age-Related Cognitive Deficits and Down-Regulates Alzheimer's Markers in SAMP8 Mice	29346315
Diverse Brain Myeloid Expression Profiles Reveal Distinct Microglial Activation States and Aspects of Alzheimer's Disease Not Evident in Mouse Models	29346778
Molecular and functional signatures in a novel Alzheimer's disease mouse model assessed by quantitative proteomics	29338754
Schisandrin ameliorates cognitive impairment and attenuates Aβ deposition in APP/PS1 transgenic mice: involvement of adjusting neurotransmitters and their metabolite changes in the brain	29323336
Endoplasmic reticulum stress responses in mouse models of Alzheimer's disease: Overexpression paradigm versus knockin paradigm	29298895
The effects of exercise on hypothalamic neurodegeneration of Alzheimer's disease mouse model	29293568
Increased Insoluble Amyloid-β Induces Negligible Cognitive Deficits in Old AppNL/NL Knock-In Mice	30320577
Yokukansan Ameliorates Hippocampus-Dependent Learning Impairment in Senescence-Accelerated Mouse	30270329

Anti-α4β1 Integrin Antibodies Attenuated Brain Inflammatory Changes in a Mouse Model of Alzheimer's Disease	30068274
Walnut Supplementation in the Diet Reduces Oxidative Damage and Improves Antioxidant Status in Transgenic Mouse Model of Alzheimer's Disease	30040727
Tocotrienol-Rich Fraction of Palm Oil Improves Behavioral Impairments and Regulates Metabolic Pathways in AβPP/PS1 Mice	29889072
Banxia Xiexin decoction ameliorated cognition via the regulation of insulin pathways and glucose transporters in the hippocampus of APPswe/PS1dE9 mice	29873261
The short-term improvements of enriched environment in behaviors and pathological changes of APP/PS1 mice via regulating cytokines	29708824
Upregulation of TREM2 Ameliorates Neuroinflammatory Responses and Improves Cognitive Deficits Triggered by Surgical Trauma in Appswe/PS1dE9 Mice	29689568
Comprehensive Characterization of the Pyroglutamate Amyloid-β Induced Motor Neurodegenerative Phenotype of TBA2.1 Mice	29578479
Expression Profiling of Cytokine, Cholinergic Markers, and Amyloid-β Deposition in the APPSWE/PS1dE9 Mouse Model of Alzheimer's Disease Pathology	29439355
Increased Vulnerability of the Hippocampus in Transgenic Mice Overexpressing APP and Triple Repeat Tau	29332037
The diphenylpyrazole compound anle138b blocks Aβ channels and rescues disease phenotypes in a mouse model for amyloid pathology	29208638
Differences in Synaptic Dysfunction Between rTg4510 and APP/PS1 Mouse Models of Alzheimer's Disease	29154272
Reduced blood-brain barrier expression of fatty acid-binding protein 5 is associated with increased vulnerability of APP/PS1 mice to cognitive deficits from low omega-3 fatty acid diets	29105065
Hippocampal phosphorylated tau induced cognitive decline, dendritic spine loss and mitochondrial abnormalities in a mouse model of Alzheimer's disease	29040533
Enriched physical environment reverses spatial cognitive impairment of socially isolated APPswe/PS1dE9 transgenic mice before amyloidosis onset	29274291
An exploratory investigation of brain-selective estrogen treatment in males using a mouse model of Alzheimer's disease	29183688
Investigation of the neuroprotective effects of Lycium barbarum water extract in apoptotic cells and Alzheimer's disease mice	29257339
Calmodulin-like skin protein protects against spatial learning impairment in a mouse model of Alzheimer disease	29164613
Discrete Pools of Oligomeric Amyloid-β Track with Spatial Learning Deficits in a Mouse Model of Alzheimer Amyloidosis	29248459
Longitudinal investigation of neuroinflammation and metabolite profiles in the APP(swe) ×PS1(Δe9) transgenic mouse model of Alzheimer's disease	29124761
Seed-induced Aβ deposition is modulated by microglia under environmental enrichment in a mouse model of Alzheimer's disease	29229786
Synthesis and in vitro characterization of a P2X7 radioligand [(123)I]TZ6019 and its response to neuroinflammation in a mouse model of Alzheimer disease	29225193

In vivo and ex vivo analyses of amyloid toxicity in the Tc1 mouse model of Down syndrome	29215943
Establishment of an Alzheimer's disease model with latent herpesvirus infection using PS2 and Tg2576 double transgenic mice	29187699
Disease-modifying benefit of Fyn blockade persists after washout in mouse Alzheimer's model	29191754
Detection of β-Amyloid by Sialic Acid Coated Bovine Serum Albumin Magnetic Nanoparticles in a Mouse Model of Alzheimer's Disease	29134771
Magnesium boosts the memory restorative effect of environmental enrichment in Alzheimer's disease mice	29125684
Age-related epigenetic changes in hippocampal subregions of four animal models of Alzheimer's disease	29113959
Brain 5-lipoxygenase over-expression worsens memory, synaptic integrity, and tau pathology in the P301S mice	29106033
Resveratrol promotes hUC-MSCs engraftment and neural repair in a mouse model of Alzheimer's disease	29102593
The hypothalamus as the primary brain region of metabolic abnormalities in APP/PS1 transgenic mouse model of Alzheimer's disease	29107091
Neuroinflammation Appears Early on PET Imaging and Then Plateaus in a Mouse Model of Alzheimer Disease	28986511
Atypical PKC, PKCλ/ι, activates β-secretase and increases Aβ(1-40/42) and phospho-tau in mouse brain and isolated neuronal cells, and may link hyperinsulinemia and other aPKC activators to development of pathological and memory abnormalities in Alzheimer's disease	29032894
Increased astroglial activity and reduced neuronal function across brain in AβPP-PS1 mouse model of Alzheimer's disease	28585882
β-Amyloid Precursor Protein Intracellular Domain Controls Mitochondrial Function by Modulating Phosphatase and Tensin Homolog-Induced Kinase 1 Transcription in Cells and in Alzheimer Mice Models	28587718
Alzheimer mouse brain tissue measured by time resolved fluorescence spectroscopy using single- and multi-photon excitation of label free native molecules	28464457
NLRP3 Inflammasome Inhibitor Ameliorates Amyloid Pathology in a Mouse Model of Alzheimer's Disease	28255908
