# Supplementary material for: What’s not in the news headlines or titles of Alzheimer disease articles? #InMice
Source: PLoS Biol. 2021 Jun 15;19(6):e3001260. doi: 10.1371/journal.pbio.3001260 (PMC8205157; doi:10.1371/journal.pbio.3001260)
Supplement: S2 Table — (PDF) [file pbio.3001260.s002.pdf]

S2 Table: List of Nondeclarative Papers (N=218)

Title	*PMID*
Targeting soluble tumor necrosis factor as a potential intervention to lower risk for late-onset Alzheimer's disease associated with obesity, metabolic syndrome, and type 2 diabetes	31892368
A small molecule transcription factor EB activator ameliorates beta-amyloid precursor protein and Tau pathology in Alzheimer's disease models	31858697
Genetic deletion of soluble epoxide hydrolase delays the progression of Alzheimer's disease	31847859
FA-97, a New Synthetic Caffeic Acid Phenethyl Ester Derivative, Protects against Oxidative Stress-Mediated Neuronal Cell Apoptosis and Scopolamine-Induced Cognitive Impairment by Activating Nrf2/HO-1 Signaling	31885818
The selective GSK3 inhibitor, SAR502250, displays neuroprotective activity and attenuates behavioral impairments in models of neuropsychiatric symptoms of Alzheimer's disease in rodents	31792284
Characterization of the selective in vitro and in vivo binding properties of crenezumab to oligomeric Aβ	31787113
Neuroprotective Effect of S-trans, Trans-farnesylthiosalicylic Acid via Inhibition of RAS/ERK Pathway for the Treatment of Alzheimer's Disease	31819374
Intranasal MMI-0100 Attenuates Aβ(1-42)- and LPS-Induced Neuroinflammation and Memory Impairments via the MK2 Signaling Pathway	31849936
Elevating acetyl-CoA levels reduces aspects of brain aging	31742554
Intra- and extracellular β-amyloid overexpression via adeno-associated virus-mediated gene transfer impairs memory and synaptic plasticity in the hippocampus	31685865
Cuscutae Japonicae Semen Ameliorates Memory Dysfunction by Rescuing Synaptic Damage in Alzheimer's Disease Models	31661844
Evaluation of B(0)-correction of relative CBF maps using tagging distance dependent Z-spectrum (TADDZ)	31669538
Lack of hepatic apoE does not influence early Aβ deposition: observations from a new APOE knock-in model	31623648
Imbalance in the response of pre- and post-synaptic components to amyloidopathy	31619689
Amyloid precursor protein, an androgen-regulated gene, is targeted by RNA-binding protein PSF/SFPQ in neuronal cells	31541592
Dietary salt promotes cognitive impairment through tau phosphorylation	31645758
Cyclooxygenase-2 Induced the β-Amyloid Protein Deposition and Neuronal Apoptosis Via Upregulating the Synthesis of Prostaglandin E(2) and 15-Deoxy-Δ(12,14)-prostaglandin J(2)	31392591
Lifelong choline supplementation ameliorates Alzheimer's disease pathology and associated cognitive deficits by attenuating microglia activation	31560162
Cyclooxygenase-2 is critical for the propagation of β-amyloid protein and reducing the glycosylation of tau in Alzheimer's disease	31551514
Amyloid-Beta Modulates Low-Threshold Activated Voltage-Gated L-Type Calcium Channels of Arcuate Neuropeptide Y Neurons Leading to Calcium Dysregulation and Hypothalamic Dysfunction	31537707

Intracerebral Injection of Extracellular Vesicles from Mesenchymal Stem Cells Exerts Reduced Aβ Plaque Burden in Early Stages of a Preclinical Model of Alzheimer's Disease	31510042
L-Type Amino Acid Transporter 1 (LAT1/Lat1)-Utilizing Prodrugs Can Improve the Delivery of Drugs into Neurons, Astrocytes and Microglia	31492955
Loss in efficacy measures of tolfenamic acid in a tau knock-out model: Relevance to Alzheimer's disease	31450960
Epitope Mapping by NMR of a Novel Anti-Aβ Antibody (STAB-MAb)	31439854
Sustained microglial depletion with CSF1R inhibitor impairs parenchymal plaque development in an Alzheimer's disease model	31434879
Possible epigenetic regulatory effect of dysregulated circular RNAs in Alzheimer's disease model	31420566
Beneficial effects of curtailing immune susceptibility in an Alzheimer's disease model	31409354
Cell-autonomous and non-cell autonomous effects of neuronal BIN1 loss in vivo	31408457
A vicious cycle of β amyloid-dependent neuronal hyperactivation	31395777
Therapeutic antibody targeting microtubule-binding domain prevents neuronal internalization of extracellular tau via masking neuron surface proteoglycans	31391090
Adenovirus 36 improves glycemic control and markers of Alzheimer's disease pathogenesis	31398466
Synaptic and memory dysfunction in a β-amyloid model of early Alzheimer's disease depends on increased formation of ATP-derived extracellular adenosine	31394204
Induced neuronal activity does not attenuate amyloid beta-induced synaptic loss in vitro	31376224
Reversibility of Age-related Oxidized Free NADH Redox States in Alzheimer's Disease Neurons by Imposed External Cys/CySS Redox Shifts	31375701
Novel beta-amyloid aggregate inhibitors for Alzheimer's disease	31416983
Effects of single and combined immunotherapy approach targeting amyloid β protein and α-synuclein in a dementia with Lewy bodies-like model	31378574
Systemic inflammation impairs microglial Aβ clearance through NLRP3 inflammasome	31359456
Aspirin up-regulates suppressor of cytokine signaling 3 in glial cells via PPARα	31273781
Oxidative inactivation of amyloid beta-degrading proteases by cholesterol-enhanced mitochondrial stress	31376793
Collateral Vessels Have Unique Endothelial and Smooth Muscle Cell Phenotypes	31344780
TREM2 Acts Downstream of CD33 in Modulating Microglial Pathology in Alzheimer's Disease	31301936
Characterization of the unique In Vitro effects of unsaturated fatty acids on the formation of amyloid β fibrils	31291354
Over-expression of miR-34a induces rapid cognitive impairment and Alzheimer's disease-like pathology	31295467
Ketones improves Apolipoprotein E4-related memory deficiency via sirtuin 3	31280254
Tau pathology reduction with SM07883, a novel, potent, and selective oral DYRK1A inhibitor: A potential therapeutic for Alzheimer's disease	31267651
Copper-Induced Upregulation of MicroRNAs Directs the Suppression of Endothelial LRP1 in Alzheimer's Disease Model	30923833
Preventive Effects of Tryptophan-Methionine Dipeptide on Neural Inflammation and Alzheimer's Pathology	31261895

A Breakdown in Metabolic Reprogramming Causes Microglia Dysfunction in Alzheimer's Disease	31257151
Endothelin type B receptor promotes cofilin rod formation and dendritic loss in neurons by inducing oxidative stress and cofilin activation	31248984
Synaptic and memory dysfunction induced by tau oligomers is rescued by up-regulation of the nitric oxide cascade	31248451
Experimental evidence for the age dependence of tau protein spread in the brain	31249873
Tuning of Glutamate, But Not GABA, Release by an Intrasynaptic Vesicle APP Domain Whose Function Can Be Modulated by β- or α-Secretase Cleavage	31235642
Grape seed proanthocyanidins ameliorate neuronal oxidative damage by inhibiting GSK-3β-dependent mitochondrial permeability transition pore opening in an experimental model of sporadic Alzheimer's disease	31232699
Rosmarinic acid suppresses Alzheimer's disease development by reducing amyloid β aggregation by increasing monoamine secretion	31213631
The Alzheimer's Disease-Associated Protein BACE1 Modulates T Cell Activation and Th17 Function	31209103
The intracellular domain of CX3CL1 regulates adult neurogenesis and Alzheimer's amyloid pathology	31209068
Hippocampal stem cells promotes synaptic resistance to the dysfunctional impact of amyloid beta oligomers via secreted exosomes	31200742
ADAMTS13 maintains cerebrovascular integrity to ameliorate Alzheimer-like pathology	31185010
Novel MicroRNA-455-3p and its protective effects against abnormal APP processing and amyloid beta toxicity in Alzheimer's disease	31181293
Soluble epoxide hydrolase modulates immune responses in activated astrocytes involving regulation of STAT3 activity	31176371
Aβ oligomers trigger and accelerate Aβ seeding	31099449
Tau binding protein CAPON induces tau aggregation and neurodegeneration	31160584
Altered Cytoskeletal Composition and Delayed Neurite Elongation in tau(45-230)-Expressing Hippocampal Neurons	31158440
Opposite microglial activation stages upon loss of PGRN or TREM2 result in reduced cerebral glucose metabolism	31122931
Pharmacokinetic parameters and mechanism of action of an efficient anti-Aβ single chain antibody fragment	31150495
Tryptophan-related dipeptides in fermented dairy products suppress microglial activation and prevent cognitive decline	31121563
Rapamycin and Alzheimer disease: a double-edged sword?	31066320
A Novel Apolipoprotein E Antagonist Functionally Blocks Apolipoprotein E Interaction With N-terminal Amyloid Precursor Protein, Reduces β-Amyloid-Associated Pathology, and Improves Cognition	31208706
Astaxanthin exerts protective effects similar to bexarotene in Alzheimer's disease by modulating amyloid-beta and cholesterol homeostasis in blood-brain barrier endothelial cells	31055081
BDNF-producing, amyloid β-specific CD4 T cells as targeted drug-delivery vehicles in Alzheimer's disease	31085101

Neuroinflammation and amyloid-beta 40 are associated with reduced serotonin transporter (SERT) activity in a transgenic model of familial Alzheimer's disease	31043179
Synapse loss and progress of Alzheimer's disease -A network model	31024073
Glutamine Improves Oxidative Stress through the Wnt3a/β-Catenin Signaling Pathway in Alzheimer's Disease In Vitro and In Vivo	31119171
Aberrant Excitatory-Inhibitory Synaptic Mechanisms in Entorhinal Cortex Microcircuits During the Pathogenesis of Alzheimer's Disease	30766992
Soluble TREM2 ameliorates pathological phenotypes by modulating microglial functions in an Alzheimer's disease model	30911003
Dysfunction of the ubiquitin ligase E3A Ube3A/E6-AP contributes to synaptic pathology in Alzheimer's disease	30937395
Magnetic resonance imaging of noradrenergic neurons	30903359
Effects of Resveratrol on the Mechanisms of Antioxidants and Estrogen in Alzheimer's Disease	31016201
Multi-sensory Gamma Stimulation Ameliorates Alzheimer's-Associated Pathology and Improves Cognition	30879788
Amyloid β oligomers suppress excitatory transmitter release via presynaptic depletion of phosphatidylinositol-4,5-bisphosphate	30867420
Neuronal AMP-activated protein kinase hyper-activation induces synaptic loss by an autophagy-mediated process	30833547
PKR knockout in the 5xFAD model of Alzheimer's disease reveals beneficial effects on spatial memory and brain lesions	30821420
Sodium rutin ameliorates Alzheimer's disease-like pathology by enhancing microglial amyloid-β clearance	30820451
Chronic cerebral hypoperfusion shifts the equilibrium of amyloid β oligomers to aggregation-prone species with higher molecular weight	30808940
Systems biology identifies preserved integrity but impaired metabolism of mitochondria due to a glycolytic defect in Alzheimer's disease neurons	30793475
Myoinositol CEST signal in animals with increased Iba-1 levels in response to an inflammatory challenge-Preliminary findings	30789943
In vivo localization of human acetylcholinesterase-derived species in a β-sheet conformation at the core of senile plaques in Alzheimer's disease	30787102
APP depletion alters selective pre- and post-synaptic proteins	30763689
Hyperoxygenation revitalizes Alzheimer's disease pathology through the upregulation of neurotrophic factors	30746828
APOE4-mediated amyloid-β pathology depends on its neuronal receptor LRP1	30741718
Opposing Roles of apolipoprotein E in aging and neurodegeneration	30760557
Age- and AD-related redox state of NADH in subcellular compartments by fluorescence lifetime imaging microscopy	30729413
Activation of MT2 receptor ameliorates dendritic abnormalities in Alzheimer's disease via C/EBPα/miR-125b pathway	30706990
Inhibition of Stat3-mediated astrogliosis ameliorates pathology in an Alzheimer's disease model	30617153
Near-infrared Fluorescence Ocular Imaging (NIRFOI) of Alzheimer's Disease	29802553

Multi-faceted therapeutic strategy for treatment of Alzheimer's disease by concurrent administration of etodolac and α-tocopherol	30710675
Poly(propylene imine) dendrimers with histidine-maltose shell as novel type of nanoparticles for synapse and memory protection	30708052
Derivatives of Piperazines as Potential Therapeutic Agents for Alzheimer's Disease	30696719
A novel crosslinking protocol stabilizes amyloid β oligomers capable of inducing Alzheimer's-associated pathologies	30565253
Modernization of Golgi staining techniques for high-resolution, 3-dimensional imaging of individual neurons	30644431
Protective roles of isoastilbin against Alzheimer's disease via Nrf2-mediated antioxidation and anti-apoptosis	30664148
Rescue of Transgenic Alzheimer's Pathophysiology by Polymeric Cellular Prion Protein Antagonists	30605671
Intraneuronal Tau Misfolding Induced by Extracellular Amyloid-β Oligomers	31524157
Production of the herb Ruta chalepensis L. expressing amyloid β-GFP fusion protein	31189782
A Robust and Scalable High-Throughput Compatible Assay for Screening Amyloid-β-Binding Compounds	31177230
Locus Coeruleus Degeneration Induces Forebrain Vascular Pathology in a Transgenic Rat Model of Alzheimer's Disease	31177220
Neuroprotective Effects of the Amylin Analog, Pramlintide, on Alzheimer's Disease Are Associated with Oxidative Stress Regulation Mechanisms	30958347
CD11a expression distinguishes infiltrating myeloid cells from plaque-associated microglia in Alzheimer's disease	30588668
Adult hippocampal neurogenesis occurs in the absence of Presenilin 1 and Presenilin 2	30560948
Tau impairs neural circuits, dominating amyloid-β effects, in Alzheimer models in vivo	30559471
Protein aggregation linked to Alzheimer's disease revealed by saturation transfer MRI	30553917
Bojungikgi-Tang, a Traditional Herbal Formula, Exerts Neuroprotective Effects and Ameliorates Memory Impairments in Alzheimer's Disease-Like Experimental Models	30544702
Spatiotemporal activation of the C/EBPβ/δ-secretase axis regulates the pathogenesis of Alzheimer's disease	30530690
Alzheimer's Disease Risk Factor Pyk2 Mediates Amyloid-β-Induced Synaptic Dysfunction and Loss	30518596
Genome-wide identification of genic and intergenic neuronal DNA regions bound by Tau protein under physiological and stress conditions	30321409
Intracellular metalloprotease activity controls intraneuronal Aβ aggregation and limits secretion of Aβ via exosomes	30481490
GSK3β-mediated tau hyperphosphorylation triggers diabetic retinal neurodegeneration by disrupting synaptic and mitochondrial functions	30466464
Label-free imaging of amyloid plaques in Alzheimer's disease with stimulated Raman scattering microscopy	30456301
Presenilin-mediated cleavage of APP regulates synaptotagmin-7 and presynaptic plasticity	30429473
Protective and reversal actions of a novel peptidomimetic against a pivotal toxin implicated in Alzheimer's disease	30551355

Functional assessments through novel proteomics approaches: Application to insulin/IGF signaling in neurodegenerative disease'	30412730
Microglial response to increasing amyloid load saturates with aging: a longitudinal dual tracer in vivo μPET-study	30400912
Transferrin is responsible for mediating the effects of iron ions on the regulation of anterior pharynx-defective-1α/β and Presenilin 1 expression via PGE(2) and PGD(2) at the early stage of Alzheimer's Disease	30383537
The BACE-1 inhibitor CNP520 for prevention trials in Alzheimer's disease	30224383
Curcumin-loaded PLGA-PEG nanoparticles conjugated with B6 peptide for potential use in Alzheimer's disease	30107760
Delta-secretase (AEP) mediates tau-splicing imbalance and accelerates cognitive decline in tauopathies	30373880
Sirtuin 3 attenuates amyloid-β induced neuronal hypometabolism	30362958
APP promotes osteoblast survival and bone formation by regulating mitochondrial function and preventing oxidative stress	30349052
APOE ε2 is associated with increased tau pathology in primary tauopathy	30348994
Matured Hop-Derived Bitter Components in Beer Improve Hippocampus-Dependent Memory Through Activation of the Vagus Nerve	30337611
Transcranial optical imaging reveals a pathway for optimizing the delivery of immunotherapeutics to the brain	30333324
The cargo receptor SQSTM1 ameliorates neurofibrillary tangle pathology and spreading through selective targeting of pathological MAPT (microtubule associated protein tau)	30290707
Tau protein aggregation is associated with cellular senescence in the brain	30126037
Brain Penetrating Bifunctional Erythropoietin-Transferrin Receptor Antibody Fusion Protein for Alzheimer's Disease	30252487
Neuronal susceptibility to beta-amyloid toxicity and ischemic injury involves histone deacetylase-2 regulation of endophilin-B1	30028551
Mini-GAGR, an intranasally applied polysaccharide, activates the neuronal Nrf2-mediated antioxidant defense system	30282635
Anti-Inflammatory, Anti-Diabetic, and Anti-Alzheimer's Effects of Prenylated Flavonoids from Okinawa Propolis: An Investigation by Experimental and Computational Studies	30262742
Differential effects of partial and complete loss of TREM2 on microglial injury response and tauopathy	30232263
Thiamine deficiency contributes to synapse and neural circuit defects	30231926
Pharmacological basis for application of scutellarin in Alzheimer's disease: Antioxidation and antiapoptosis	30221730
Inhibition of hematopoietic cell kinase dysregulates microglial function and accelerates early stage Alzheimer's disease-like neuropathology	30277607
Neuronal calcineurin transcriptional targets parallel changes observed in Alzheimer disease brain	29806693
RPS23RG1 Is Required for Synaptic Integrity and Rescues Alzheimer's Disease-Associated Cognitive Deficits	30292394

PLD3 and PLD4 are single-stranded acid exonucleases that regulate endosomal nucleic-acid sensing	30111894
Functionalization of stable fluorescent nanodiamonds towards reliable detection of biomarkers for Alzheimer's disease	30097010
Microglial translational profiling reveals a convergent APOE pathway from aging, amyloid, and tau	30082275
PD-1 deficiency is not sufficient to induce myeloid mobilization to the brain or alter the inflammatory profile during chronic neurodegeneration	30086399
Highly sensitive/selective 3D nanostructured immunoparticle-based interface on a multichannel sensor array for detecting amyloid-beta in Alzheimer's disease	30128048
Functional aspects of meningeal lymphatics in ageing and Alzheimer's disease	30046111
The adhesion and migration of microglia to β-amyloid (Aβ) is decreased with aging and inhibited by Nogo/NgR pathway	30029608
CIP2A Causes Tau/APP Phosphorylation, Synaptopathy, and Memory Deficits in Alzheimer's Disease	30021167
Active PSF shaping and adaptive optics enable volumetric localization microscopy through brain sections	30013047
Modulation of the intrinsic neuronal excitability by multifunctional liposomes tailored for the treatment of Alzheimer's disease	30034232
Tetrahydroxystilbene glycoside antagonizes β-amyloid-induced inflammatory injury in microglia cells by regulating PU.1 expression	29668503
Human ApoE Isoforms Differentially Modulate Brain Glucose and Ketone Body Metabolism: Implications for Alzheimer's Disease Risk Reduction and Early Intervention	29967007
Alterations of functional circuitry in aging brain and the impact of mutated APP expression	30055413
Structural Insights of Benzenesulfonamide Analogues as NLRP3 Inflammasome Inhibitors: Design, Synthesis, and Biological Characterization	29877709
Selective targeting of 3 repeat Tau with brain penetrating single chain antibodies for the treatment of neurodegenerative disorders	29934874
Store depletion-induced h-channel plasticity rescues a channelopathy linked to Alzheimer's disease	29906573
Proteasome stress leads to APP axonal transport defects by promoting its amyloidogenic processing in lysosomes	29724915
Novel interaction between Alzheimer's disease-related protein presenilin 1 and glutamate transporter 1	29880815
Enriched Brain Omega-3 Polyunsaturated Fatty Acids Confer Neuroprotection against Microinfarction	29880270
Butterbur Leaves Attenuate Memory Impairment and Neuronal Cell Damage in Amyloid Beta-Induced Alzheimer's Disease Models	29865187
The Trem2 R47H variant confers loss-of-function-like phenotypes in Alzheimer's disease	29859094
Classification of Spatiotemporal Neural Activity Patterns in Brain Imaging Data	29844346
Protein kinase Cα gain-of-function variant in Alzheimer's disease displays enhanced catalysis by a mechanism that evades down-regulation	29844158

Piperlongumine Improves Lipopolysaccharide-Induced Amyloidogenesis by Suppressing NF-KappaB Pathway	29802525
A silver lining for 24-hydroxycholesterol in Alzheimer's disease: The involvement of the neuroprotective enzyme sirtuin 1	29883958
Pharmacological Basis for the Use of Evodiamine in Alzheimer's Disease: Antioxidation and Antiapoptosis	29883380
Identification and therapeutic modulation of a pro-inflammatory subset of disease-associated-microglia in Alzheimer's disease	29784049
Inhibition of PKCδ reduces amyloid-β levels and reverses Alzheimer disease phenotypes	29739836
Defensive effect of microRNA-200b/c against amyloid-beta peptide-induced toxicity in Alzheimer's disease models	29738527
The Medial Septum Is Insulin Resistant in the AD Presymptomatic Phase: Rescue by Nerve Growth Factor-Driven IRS(1) Activation	29736736
A self-destructive nanosweeper that captures and clears amyloid β-peptides	29728565
P2Y1 receptor blockade normalizes network dysfunction and cognition in an Alzheimer's disease model	29724785
Bitopic Binding Mode of an M(1) Muscarinic Acetylcholine Receptor Agonist Associated with Adverse Clinical Trial Outcomes	29695609
Distinct in vivo roles of secreted APP ectodomain variants APPsα and APPsβ in regulation of spine density, synaptic plasticity, and cognition	29661886
Phosphorylation of Tau protein correlates with changes in hippocampal theta oscillations and reduces hippocampal excitability in Alzheimer's model	29632073
SUMO1-conjugation is altered during normal aging but not by increased amyloid burden	29633471
Asparagus cochinchinensis stimulates release of nerve growth factor and abrogates oxidative stress in the Tg2576 model for Alzheimer's disease	29625607
Interaction between a MAPT variant causing frontotemporal dementia and mutant APP affects axonal transport	29729423
Altered DNA repair; an early pathogenic pathway in Alzheimer's disease and obesity	29618789
Novel monoclonal antibodies targeting the microtubule-binding domain of human tau	29608591
Innate immune memory in the brain shapes neurological disease hallmarks	29643512
Targeting of nonlipidated, aggregated apoE with antibodies inhibits amyloid accumulation	29600961
Amyloid-beta modulates microglial responses by binding to the triggering receptor expressed on myeloid cells 2 (TREM2)	29587871
Memantine loaded PLGA PEGylated nanoparticles for Alzheimer's disease: in vitro and in vivo characterization	29587747
Characterization of Hit Compounds Identified from High-throughput Screening for their Effect on Blood-brain Barrier Integrity and Amyloid-β Clearance: In Vitro and In Vivo Studies	29596966
BACE1 SUMOylation increases its stability and escalates the protease activity in Alzheimer's disease	29581300
nNOS-CAPON interaction mediates amyloid-β-induced neurotoxicity, especially in the early stages	29577585
Homocysteine Increases Tau Phosphorylation, Truncation and Oligomerization	29562600

Paper-based electrochemiluminescence sensor for highly sensitive detection of amyloid-β oligomerization: Toward potential diagnosis of Alzheimer's disease	29721080
Elevated TREM2 Gene Dosage Reprograms Microglia Responsivity and Ameliorates Pathological Phenotypes in Alzheimer's Disease Models	29518357
TREM2 Is a Receptor for β-Amyloid that Mediates Microglial Function	29518356
Differential Phagocytic Properties of CD45(low) Microglia and CD45(high) Brain Mononuclear Phagocytes-Activation and Age-Related Effects	29552013
ApoE facilitates the microglial response to amyloid plaque pathology	29483128
Involvement of impaired autophagy and mitophagy in Neuro-2a cell damage under hypoxic and/or high-glucose conditions	29459731
A role for tau in learning, memory and synaptic plasticity	29453339
The Mechanisms of Bushen-Yizhi Formula as a Therapeutic Agent against Alzheimer's Disease	29449587
The APPswe/PS1A246E mutations in an astrocytic cell line leads to increased vulnerability to oxygen and glucose deprivation, Ca(2+) dysregulation, and mitochondrial abnormalities	29315575
Guanosine monophosphate reductase 1 is a potential therapeutic target for Alzheimer's disease	29426890
Mfn2 ablation causes an oxidative stress response and eventual neuronal death in the hippocampus and cortex	29391029
Intraventricular infusion of clusterin ameliorated cognition and pathology in Tg6799 model of Alzheimer's disease	29370749
Candesartan ameliorates brain inflammation associated with Alzheimer's disease	29365370
Stereochemistry and amyloid inhibition: Asymmetric triplex metallohelices enantioselectively bind to Aβ peptide	29372182
Cromolyn Reduces Levels of the Alzheimer's Disease-Associated Amyloid β-Protein by Promoting Microglial Phagocytosis	29348604
Impact of Swiprosin-1/Efhd2 on Adult Hippocampal Neurogenesis	29337116
Brain Shuttle Antibody for Alzheimer's Disease with Attenuated Peripheral Effector Function due to an Inverted Binding Mode	29298417
The prodrug of 7,8-dihydroxyflavone development and therapeutic efficacy for treating Alzheimer's disease	29295929
Migration-based selections of antibodies that convert bone marrow into trafficking microglia-like cells that reduce brain amyloid β	29295920
The Stress-Induced Transcription Factor NR4A1 Adjusts Mitochondrial Function and Synapse Number in Prefrontal Cortex	29295823
Protective Effects of 2-Dodecyl-6-Methoxycyclohexa-2,5 -Diene-1,4-Dione Isolated from Averrhoa Carambola L. (Oxalidaceae) Roots on Neuron Apoptosis and Memory Deficits in Alzheimer's Disease	30196278
Pathological missorting of endogenous MAPT/Tau in neurons caused by failure of protein degradation systems	30145931
Involvement of Activation of Asparaginyl Endopeptidase in Tau Hyperphosphorylation in Repetitive Mild Traumatic Brain Injury	29889065
Bajijiasu Ameliorates β-Amyloid-Triggered Endoplasmic Reticulum Stress and Related Pathologies in an Alzheimer's Disease Model	29587274

A Novel Therapeutic Approach to Treat Alzheimer's Disease by Neurotrophic Support During the Period of Synaptic Compensation	29562539
Inhibition of mTOR protects the blood-brain barrier in models of Alzheimer's disease and vascular cognitive impairment	29351469
Nrf2 mediates the expression of BAG3 and autophagy cargo adaptor proteins and tau clearance in an age-dependent manner	29304346
