# Supplementary material for: What’s not in the news headlines or titles of Alzheimer disease articles? #InMice
Source: PLoS Biol. 2021 Jun 15;19(6):e3001260. doi: 10.1371/journal.pbio.3001260 (PMC8205157; doi:10.1371/journal.pbio.3001260)
Supplement: S3 Table — (PDF) [file pbio.3001260.s003.pdf]

S3 Table: List of Journals and their Guidelines for Papers' Titles (N=156)

Journal	Does the journal require the study's species to be informed in the title?	Does the journal limit the number of words or characters in the title?
**ACS Chemical Neuroscience**	no	no
**Acta Neuropathologica**	no	no
**Acta Neuropathologica Communications**	no	no
**Acta Pharmacologica Sinica**	no	< 120 characters
**Acupuncture in Medicine**	no	no
**Aging**	no	no
**Aging Cell**	no	no
**Alzheimer's & Dementia: the Journal of the Alzheimer's Association**	no	no
**Alzheimer's Research & Therapy**	no	no
**American Journal of Pathology**	no	no
**Annals of Clinical and Translational Neurology**	no	100 characters max
**ASN Neuro**	no	no
**Autophagy**	no	no
**BBA - Molecular Basis of Disease**	no	no
**Behavioural Neurology**	no	no
**Biochemical Pharmacology**	no	no
**Biological and Pharmaceutical Bulletin**	no	no
**Biological Psychiatry**	no	200 characters max
**Biological Research**	no	no
**BioMed Research International**	no	no
**Biomedicine & Pharmacotherapy**	no	no
**Biomolecules**	yes	no
**Bioscience Reports**	no	20 words max
**BMC Anesthesiology**	no	no
**BMC Complementary and Alternative Medicine**	no	no
**BMC Immunology**	no	no
**BMC Neuroscience**	no	no
**Brain and Behavior**	no	no
**Brain Pathology**	no	no
**Brain Research**	no	no
**Brain, Behavior & Immunity**	no	no
**Brain: A Journal of Neurology**	no	100 characters max
**British Journal of Pharmacology**	no	160 characters max
**Cell**	no	150 characters max
**Cell Chemical Biology**	no	150 characters max
**Cell Death & Disease**	no	< 120 characters max

**Cell Reports**	no	150 Characters max
**Cell Transplantation**	no	no
**Cells**	yes	no
**Cellular & Molecular Immunology**	no	150 characters max
**Cerebral Cortex**	no	no
**Clinical Interventions in Aging**	no	no
**CNS Neuroscience & Therapeutics**	no	no
**Communications Biology**	no	< 15 words
**Critical Care**	no	no
**Current Alzheimer Research**	no	120 characters max
**Drug Delivery**	no	no
**Drug Design, Development and Therapy**	no	no
**EBioMedicine**	no	no
**eLife**	no	no
**EMBO Journal**	no	no
**EMBO Molecular Medicine**	no	no
**eNeuro**	no	no
**Epigenomics**	no	120 charcaters max
**European Journal of Neuroscience**	no	no
**European review for medical and pharmacological sciences**	no	no
**Experimental and Molecular Medicine**	no	150 characters max
**Experimental Biology and Medicine**	no	no
**FASEB Journal**	no	no
**Frontiers in immunology**	no	no
**Genes to Cells**	no	< 145 characters
**GeroScience**	no	no
**Glia**	no	no
**Hong Kong Medical Journal**	no	no
**Human Molecular Genetics**	no	no
**Human vaccines immunotherapeutics**	no	no
**International Journal of Biological Macromolecules**	no	no
**International Journal of Biological Sciences**	no	no
**International Journal of Immunopathology and Pharmacology**	no	no
**International Journal of Molecular Medicine**	no	no
**International Journal of Molecular Sciences**	yes	no
**International Journal of Nanomedicine**	no	no
**Investigative Ophthalmology & Visual Science**	no	150 characters max
**JCI Insight**	no	15 words max
**Journal of Alzheimer's Disease**	no	no
**Journal of Anatomy**	no	no
**Journal of Biological Chemistry**	no	150 characters max

**Journal of Biomedical Optics**	no	no
**Journal of Biophotonics**	no	150 characters max
**Journal of Biosciences**	no	no
**Journal of Cell Science**	no	120 characters max
**Journal of Cerebral Blood Flow & Metabolism**	no	no
**Journal of Clinical Investigation**	no	15 words max
**Journal of Controlled Release**	no	no
**Journal of Nanobiotechnology**	no	no
**Journal of Neurochemistry**	no	no
**Journal of Neuroimmunology**	no	no
**Journal of Neuroinflammation**	no	no
**Journal of Neuroscience**	no	50 words max
**Journal of Neuroscience Methods**	no	no
**Journal of Nuclear Medicine**	no	200 characters max
**Journal of Stroke & Cerebrovascular Diseases**	no	no
**Journal of the American College of Cardiology**	no	no
**Journal of traditional Chinese medicine**	no	no
**Journals of Gerontology Series A: Biological Sciences & Medical Sciences**	no	no
**Life Science Alliance**	no	100 characters max
**Life Sciences**	no	no
**Magnetic Resonance Imaging**	no	no
**Marine Drugs**	yes	no
**MCN: Molecular & Cellular Neuroscience**	no	no
**Medical science monitor international medical journal of experimental and clinical research**	no	no
**Molecular Brain**	no	no
**Molecular Medicine Reports**	no	no
**Molecular Metabolism**	no	no
**Molecular Neurobiology**	no	no
**Molecular Neurodegeneration**	no	no
**Molecular Pharmaceutics**	no	no
**Molecular Pharmacology**	no	no
**Molecular Psychiatry**	no	75 characters max
**Molecules**	yes	no
**Nature**	no	75 characters max
**Nature Communications**	no	15 words max
**Nature Immunology**	no	no
**Nature Methods**	no	no
**Nature Neuroscience SAIU**	no	no
**Naunyn-Schmiedeberg's Archives of Pharmacology**	no	no
**Neural Plasticity**	no	no

**Neurobiology of Aging**	no	no
**Neurobiology of Disease**	no	no
**Neurobiology of Learning & Memory**	no	no
**Neurochemical Research**	no	no
**NeuroImage**	no	no
**NeuroImage: Clinical**	no	no
**NeuroMolecular Medicine**	no	no
**Neuron**	no	150 characters max
**Neuropharmacology**	no	no
**Neuropsychopharmacology Reports**	no	no
**NeuroReport**	no	no
**Neuroscience**	no	no
**Neuroscience Bulletin**	no	no
**Neuroscience Letters**	no	no
**Neurotherapeutics**	no	no
**Nuclear Medicine & Biology**	no	no
**Nucleic Acids Research**	no	no
**Nutrients**	yes	no
**Oxidative Medicine & Cellular Longetivity**	no	no
**Particle and Fibre Toxicology**	no	no
**Physiological Reports**	yes	160 characters max
**Physiological Research**	no	no
**PLoS Biology**	no	250 characters max
**PLoS Genetics**	no	200 characters max
**PLoS ONE**	no	no
**PLoS Pathogens**	no	200 characters max
**Proceedings of the Japan Academy, Series B: Physical and Biological Sciences**	no	no
**Proceedings of the National Academy of Sciences of the United States of America**	yes	< 135 characters
**Psychopharmacology**	no	no
**Redox Biology**	no	no
**Science**	no	95 characters max
**Science Advances**	no	135 characters max
**Scientific Reports**	no	20 words max
**The Anatomical Record: Advances in Integrative Anatomy and Evolutionary Biology**	no	no
**The Journal of Experimental Medicine**	no	100 characters max
**The Journal of Immunology**	no	no
**Theranostics**	no	no
**Toxicological Sciences**	no	no
**Translational Psychiatry**	no	150 characters max
