# Supplementary material for: What’s not in the news headlines or titles of Alzheimer disease articles? #InMice
Source: PLoS Biol. 2021 Jun 15;19(6):e3001260. doi: 10.1371/journal.pbio.3001260 (PMC8205157; doi:10.1371/journal.pbio.3001260)
Supplement: Attachment — Submitted filename: Point-by-pont.pdf [file pbio.3001260.s004.pdf]

Point-by-point
Answering reviewer #1

1.
My major concern with this manuscript, is that the discussion is a large over reach for the methods and data presented and arguably the authors could be accused of the same intention to sensationalise their outcomes that the AD community of 2018 are accused of.

Our answer:
We have significantly improved the discussion of our manuscript that now better focuses on the results we found in our study. We agree that in our previous version our discussion had some speculation, that is significantly reduced in this new version.
We are sorry if in our previous version it sounded like we were accusing the researchers of sensationalize their outcomes, this was not our intention.

2.
At best, this is a thought provoking prod at the quality of titles used in a small subset of studies in a niche area of animal models. The data is no doubt interesting but is more hypothesis generating than testing. For example, the assumption is that the title is all that is important, and fair enough, the authors provide a reference to suggest the title is all that some people read. However, there was no attempt by the authors to further test the quality of media reporting by actually reading and providing an analysis of the content of the article. The bigger question to ask here, in testing the need for better regulation on titles in studies is whether or not the totality of reporting is omitting the use of animals. Many readers of lay articles could feasibly be hooked by the title, find very quickly that the article is reporting a study in animals and either read on or not. In the way that the current manuscript is presented I find the analysis having stopped solely at title a significant flaw in the novelty and significance of the manuscript. That is to say, I had not been convinced by the authors of this manuscript that the title, independent of a complete article is a significant issue to be concerned about.

Our answer:
Our primary intention with this study was to test whether an association exists between articles' titles and news headlines regarding the mention, or not, of mice. Our intention was never to analyze the quality of the media reporting but whether media reporting was "influenced" by how the study is reported, when it comes to the article's title. Therefore, analyzing whether or not the media reporting informs in the main text that the study was done in animals was not our goal.

In this version we better set the foundations to show that most online users do not read through science news or the tweets they share. According to a survey done by the Pew Research Center on Science News and Information in 2017, only half of social media users say they click through science news stories. Of these, 10% do it often, 43% do it sometimes, and the remaining do it hardly, never or simply do not see science news stories. As for tweets, a recent study shows that 59% of the links on news stories shared on Twitter are never read (all this information was added to our discussions). Therefore, the reviewer's belief that "many readers of lay articles could feasibly be hooked by the title, find very quickly that the article is reporting a study in animals and either read on or not." does not seem to reflect what studies in the field show. If readers do not read through the news and if the news headline omits that the

study was done in animals, then readers cannot "find very quickly that the article is reporting a study in animals". We hope that this new data from the Pew Research Center on Science News and Information convinces the reviewer that most people read only the headlines.

Of note, what is the impression left on a reader that only reads the following headlines? "An Hour of Light and Sound a Day Might Keep Alzheimer's at Bay" or "How Flashing Lights Could Treat Alzheimer's Disease" ?

These news stories were published in highly regarded news outlets, namely Scientific American and BBC. The corresponding research paper's title is "Multi-sensory gamma stimulation ameliorates Alzheimer's-associated pathology and improves cognition", which according to our data could have had an impact on how these headlines were written had the authors of the study had added "in mice" to the title of their paper.

If the headline is not accurate and gives the impression that results were found in humans, when they actually apply to mice, we can't see how this is not a significant problem on how science is reported and communicated to the public.

3.Given the small number of studies identified, this manuscript could have been significantly enhanced by merely emailing the authors of the identified articles and asking them why they omitted or declared the animal species. Whether or not all replied is not an issue, but atleast some more considered primary data could have been used to support or refute the speculation provided in the current discussion.

Our answer:
We agreed with the reviewer that our original sample was small and decided to expand it. Thus, besides studies published in 2018 we included in our sample studies published in 2019; now our sample sums up 660 research articles, which has more statistical power than the sample we worked with before. We preferred to adopt this strategy than contacting researchers (as suggested by the reviewer), which in our experience with previous studies is not a successful approach as the vast majority do not reply to this type of request.

4. There is also no consideration on the "why" a primary study gets reported in the media, and this is an important question to ask given the authors make inferences that animal species is omitted to gain more hits and attention. By raising this point, the authors identify a significant limitation - there is no consideration of why or how a specific primary study reaches lay media. Knowing this is important information given that certain institutions and authors are likely to predominate lay media on the basis of reputation and output, and as such a common culture could be influencing the study data here, beyond the limited considerations (such as spin) presented here.

Our answer:
We did not investigate this issue further because we did not find any significant difference in the number of papers that generated news stories between groups. Also, in both groups of papers, most papers generated at least one news stories (77% in the declarative group and 81% in the nondeclarative group of papers) thus, most studies in both groups reached lay

media. Therefore, the question "why or how a specific primary study reaches lay media " does not seem to apply here, considering that most studies in both groups did reach lay media. However, we did find a difference between groups for the number of news stories generated and for the number of tweets. And this is precisely our point: if papers in both groups "have what it takes" to attract media attention, why one group produces significantly more news than the other (p<0.01)? According to our data, it is the fact that mice is not mentioned in the study's title that leads to more news stories and more tweets.
However, not that his is not our primarily finding and that it's possible that factors other than the omission, or not, of mice in the title may play a role on how many news stories and tweets a scientific paper generates.

5. Further, there was no consideration in the discussion provided to the contradictory result in which lay media declared animal species (n=38) despite originating from non-declared studies. Ignoring this outcome is akin to selective reporting. The authors need to be careful of accusing researchers of a malpractice if their approach itself can be questioned. For my read, the presence of this contradictory result (to the authors premise) is an important one to explore and provides evidence that there is more in the translation of a research paper title to the lay media title that needs to be explored. What is the role of the journalist here? Why did some include the animal species when the original study did not? I am not suggesting that the authors have to explore this point as a dataset to present, but there does need to be more adequate representation / discussion of this result in the discussion.

Our answer:
In this updated version of our article, we expanded the discussion of this topic. We closely examined the 70 articles in the nondeclarative group of papers that generated news stories that mentioned mice in their headlines, despite the original omission in the title of the papers these news stories report on. As an attempt to explain this finding, we first raised the hypothesis that a press release in Eurekalert (the main repository for science-related press releases) could be having an influence on the headline of news stories. Our belief was that if a press release mentioned mice in its headlines, other news stories reporting on the same scientific paper would also mention mice in their headlines. However, we found that only one Eurekalert press release was produced for a single paper among all the 70 nondeclarative research papers. We then asked if these 70 research papers had any particularity that could have driven writers to write headlines that mentioned mice. However, we found that these research papers generated both news stories omitting mice in their headlines as well as others acknowledging it. An initial analysis of the news outlets that produced news stories headlines that mentioned mice did not reveal a pattern indicating that omitting, or not, mice in the headline was an editorial decision. We also examined these 70 research articles and did not find a pattern that could explain why these research articles generated news stories with headlines acknowledging mice. Thus, we do not have an explanation for this finding at this point. No doubt this observation deserves further investigation.

As of accusing researchers of a malpractice, this was never our intention and we hope this version of our study does not pass this impression.

6. Finally, there is little if any recognition of what / who determines the title of their paper. That is disappointing given that is the cornerstone on which this work has been completed. We are given, as readers some speculation of spin and intentional deception by authors to gain more significance for their work, but for the most part, title content is determined at the journal level. Authors are provided a word or character limit, some journals will specify species identification is necessary. The authors of this manuscript could have value added to this work by reviewing the author guidelines of the journals included in their analysis to present an interesting discussion regarding whether or not, the title of published papers was adequately peer reviewed against journal requirements, or if journals themselves are the better target for regulation.

Our answer:
We truly appreciate this suggestion and decided to check the guidelines of each of the 156 journals in which at least one of the papers in our sample was published. We found that only eight journals request that authors mention the study's species in the title. We found that only 23 articles in the declarative group were published in these journals. Thus, for most papers in this group (295 - 232 = 272), acknowledging the studies' species in the title was not a decision imposed by journals' requirements. We also checked how many articles suffered any constrain in terms of limited number of either words or characters the title could have. We did not find any significant difference between groups for the number of papers published in such journals that could explain why some authors do mention mice in their articles' titles while others do not. This analysis is also explained in the Results section.

7. Currently the authors conclude that an outcome of this work is revision of the ARRIVE guidelines to include a specification of
species in title. However in the most recent ARRIVE 2.0 guideline, "title" is not identified as an item for review having been deleted from the original 2010 ARRIVE guidelines. A stronger argument could be built with a more considered investigation of the accuracy of reporting by the studies in this manuscript against the journal author guidelines.

Our answer:
Our understanding is that adding the study species to the title was never a requirement of the ARRIVE guidelines. Our belief is based on the 2010 ARRIVE and the 2.0 updated version. To our knowledge, neither version made this request.
As shown here, very few journals request that authors add the study's species to the paper's title, even though most books and guidelines on how to write a title of a scientific paper advise authors that the species used in the study should be informed in the article's title when these are not humans. We found that even when journals do request that the study's species is added to the title, they do not really enforce it. For instance, we found that 11 articles in the non-declarative group of papers were published in journals that did require the species to be added to the title.
We believe that our study offers solid evidence to justify an update to the ARRIVE guidelines.

Answering reviewer #2

1.This is an interesting study and highlights important issues for science communication. I think the paper could be strengthened if these implications for science communication more generally

were identified at the outset (ie the consequences of this specific type of 'spin'). The paper also needs some revisions in terms of reporting and particularly, the outcome reporting.

Our answer:
We produced an entire new version of this paper and we hope this version does a better job on reporting our findings.

2. In stating the research question, throughout the paper, including in the Abstract, Author Summary and Introduction, it is not completely clear what the authors actually measured. Although the authors state they measure an association, the outcome is unclear and seems to suggest they measured concordance/discordance. Simply stating the researchers investigated "whether a relationship exists" is vague; as the papers and news articles report on the same study, of course they are related. At the end of the introduction the authors state, "Our main interest was in determining whether the news headlines and the research papers' titles they refer to follow the same pattern regarding the omission, or not, of mice." This should be reflected throughout the paper and might better be described as consistency or concordance in reporting. Similarly, when reporting the findings, be consistent with your language and describe this as concordant reporting or consistent reporting rather than "media perception" (line 105), which is something entirely different.

Our answer:
In this updated version we tried to make it cleaer what we were testing in this study. To this end, we reformulated the way we presented our research question that now reads as follows: "Here we tested the hypothesis that research papers that used mice as the main study subject but omit in their titles that findings apply to mice, as opposed to humans, generate significantly more news stories with headlines that likewise omit mice, if compared to research papers with titles that mention mice."
Our research question is presented in the paragraph that starts in line 108. We no longer used the term media perception.

3. I found the Results section difficult to follow. It was often difficult to understand what exactly was measured. Often I didn't understand the distinction between one group of results and the next as it was difficult to ascertain what was being compared. I think it would help to consistently report denominators and proportions alongside numerators and to re-organize the section so that results for the full sample are clearly reported, followed by sub-group analyses (I couldn't quite tell if this occurred), sensitivity analyses (excluding titles copied verbatim), and then secondary outcome (tweets). For example, I couldn't tell what the findings on line 150-151 referred to ("Of the 853 total news pieces generated from research papers in both groups, only 229 (26.8%) were declarative, while 624 (73.2%) omitted this fact from their headlines."

Our answer:
We totally agree with the reviewer that our previous version was confuse and hard to follow. As already mentioned, we built an entire new version, produced one more image and re-wrote the Results section, providing all numbers and proportions when appropriate. We believe that with this new version the reviewer will have no difficulties to follow each of the sections, and analysis, we performed.

4. The Materials and Methods section could be strengthened. I would suggest the use of sub-headings including: Study design; Research questions and hypotheses; Data extraction; and Statistical Analysis. Key aspects that are missing are description of the outcome measures. This is a weakness throughout the paper and should be very clearly stated in the Methods, with corresponding analyses detailed.

Our answer:
As suggested, we created sub-sections in our Material and Methods section. Now the section is divided in Study design, Data extraction, and statistical analysis.
As for better description of outcome measures, we did not do any intervention in this study, so we do not understand when the reviewer asks us to add outcome measures description. We apologize.

5. Further details re: who did the screening and whether it was done in duplicate would be useful. A Prisma-type flow diagram would be very useful to summarize the sampling and reasons for exclusion.

Our answer:
The screening of papers was done by one of the authors and this information was added to the Material and Methods section.
As for the reasons for excluding papers in the nondeclarative group, virtually all papers were excluded because they also used human material such as ex-vivo, cells or even patients' samples, in the study. We wanted our sample of nondeclarative papers to include studies that worked only with mice. Because virtually all papers were excluded for this reason we did not any reason to add more details on this.

6.A limitation is that this is a convenience sample, however, I wonder if open access articles are more likely to be picked up by journalists? Is there any evidence for this?

Our answer:
We can't answer whether open access journals are more likely to be picked up by journalists than subscription-required journals. However, because all papers in both groups were published in open-access journals, any potential difference regarding the type of access does not seem to affect our analysis.

7. Please avoid all non-standard acronyms including AD, non-GM - please spell out.

Our answer:
AD refers to Alzheimer Disease and is considered a very standard acronym, so we decided to keep it throughout our text. As for non-GM, we did replace it for non-genetically modified (lane 86).

8. Could you avoid using NONDECLAPAPERS and DECLAPAPERS and simply describe the groups as you did in the Introduction? Or come up with more reader-friendly terminology?

Our answer:

In this new version we no longer use these names, we simply use declarative group and nondeclarative group. In any case, in most cases we just describe again what each group is, as suggested by the reviewer.

9. Specific comments:
Introduction, first sentence: Not just scientists are concerned and in fact, scientists sometimes the source of hype/spin

Our answer:
This sentence now is in line 97. If we agree that omitting mice from study's title is indeed a spin then our study shows that scientists, in this case, are the source for this spin, as suggested by the reviewer.

10.
By line 90-91 on page 2, I came to understand your point about the appropriateness of mouse models for understanding Alzheimer's disease, however, this understanding would have been more helpful to have up around line 52-53. I would suggest stating this main point (ie Alzheimer's Disease is a human condition; scientists have created mouse models to attempt to study the disease; but, animal models have poor predictive value) following the research aim/question and then, walking the reader through the explanations. I am not a biologist, so the explanations were most helpful.

Our answer:
We have changed the order we present the information in the introduction and we now start our text by explaining Alzheimer's Disease and animal models. Hope it's clearer now.

11.
The sentences in the abstract, author summary and the introduction are sometimes quite long and could be broken up for readability (e.g. first sentence of the Introduction, lines 105-109).

Our answer:
We made significant changes to the text and this sentence is no longer included in the text.

12.
On line 94, what is the significance of the clause "what the studies' authors consider to be AD" - could you explain?

Our answer:
We made significant changes to the text and this sentence is no longer included in the text.

13.
Your data availability statement suggests the data are proprietary/copyrighted, but the Methods on 98-100 suggest this platform is open access. Can you explain further how these data were accessed and if public, why the raw data cannot be made public? Further, if they are news headlines, are these data already in the public domain?

Our answer:

The access to Altmetric Explorer is limited for research purposes and only a limited version is offered as open access. Also, we consulted with Altmetric if the data we extracted to carry out this study could be shared with readers, but they told us they have third party subscription agreements and thus the data cannot be openly shared. We apologize for this inconvenience, but it is not our choice. Nevertheless, the entire data is fully available to you.

14.
You report "a declarative title does not impact media interest in the study." To avoid the suggestion of causality or directionality, please simply state that there was no difference between the groups.

Our answer:
We removed this sentence from the text.

15.
This sentence strikes me as belonging in the Discussion "This finding indicates that when authors openly acknowledge the use of mice, or equivalent qualifying term, in the title of their research article, writers follow the same pattern when crafting the headlines for their news stories."

Our answer
This sentence no longer belongs in the introduction.

16.
The Discussion feels a bit untethered from the study's findings at several points. I would suggest re-organizing to clearly summarize the study's key findings. Then, place each finding in the context of the wider literature in each paragraph. The discussion on the appropriateness of animal models for Alzheimer's research is interesting, however, doesn't really reflect the study's findings, so could perhaps be mentioned more succinctly. The literature on spin and the recommendations regarding reporting guidelines seem more relevant, but should be linked to specific findings.

Our answer
In this new version we better focused on our methods and results. Most of the discussion on the use of animal models to study Alzheimer's Disease was removed. We also hope to have better set the bases for considering the omission of animals in the study's title as a type of spin that is associated with more news stories and tweets.
We also re-organized both the Results section and the Discussion and we hope that each fining is better discussed in the context of the available literature.

17.
The Figures are engaging, but particularly in Figure 2, I can't understand what the final row of News articles refers to.

Our answer
We believe you are referring to Figure 1, as Figure 2 in the previous version referred to the number of tweets.

The final raw of news in Figure 1 is actually the finding that backs our hypothesis that there is an association between how authors write their papers' titles, regarding the omission, or not of mice, and how writers write their news headlines. The final row shows the numbers of news, omitting or not mice in their headlines, in each group.
