# Supplementary material for: What’s not in the news headlines or titles of Alzheimer disease articles? #InMice
Source: PLoS Biol. 2021 Jun 15;19(6):e3001260. doi: 10.1371/journal.pbio.3001260 (PMC8205157; doi:10.1371/journal.pbio.3001260)
Supplement: Attachment — Submitted filename: Point-to-point.pdf [file pbio.3001260.s005.pdf]

Answering Kieron Rooney

1. I do still have some minor issues, but I do not believe that these should prevent publication. I hope however that the authors consider in their own reflections that for my read, this paper has still not explained the why the outcome observed occurs. During the review process the authors have preferred not to interrogate further the question they raise themselves in the introduction "However, it is not known what prompts writers of news stories to either omit or knowledge (sic), in the story's headlines" This "why" this present paper raises is a more interesting question to me than the analysis presented, which I still think is more a prod than a comprehensive examination. To this end I would suggest that the authors reflect on the comment here that merely increasing the sample size as was identified in response to my query does not answer the "why does something happen", it simply increases the sample size for the "what happened". However I am content that the "why" is more a future question to ask from this work now and not a priority for the current manuscript as the major issues I had with the overreaching and speculation has been significantly dampened. In dampening the speculation, the need for a stronger mechanism / why something has happened has been reduced and this more comprehensive analysis of the outcome reported is more acceptable.
Our answer:
In the introduction of our manuscript, we do mention that it is not known what prompts writers of news stories to either omit or acknowledge mice in their stories' headlines. This study was the attempt to better understand this issue and we believe we found some important results in this regard. Our analysis reveals that writers of news stories are impacted by the way authors write their articles' titles, judging by the fact that we found a strong association between articles titles and news headlines, regarding the omission or not, of mice. To our knowledge, this is the first study to present evidence that the way science is reported by scientists plays a role on the way science news is reported by journalists.

If you ask us what motivates writers to "copycat" scientists, regarding the title of their articles/headlines, we honestly do not know. To answer this question, another study that examines the roots of science communication and journalism would be required and that would be beyond the scope of this study. Also, such analysis is not in the realm of our expertise. We sincerely hope that others will have interest in investigating further the association we present here.

To express the idea that we were in a search for finding explanations for this fact, we rewrote the fragment in the introduction to describe our hypothesis clearer, and now it reads as follows:

"It is not known what prompts writers of news stories to either omit, or acknowledge, in the story's headlines reporting on AD research, that the study was done in mice. To better understand how writers chose their news headlines in this regard, we raised the hypothesis that they are influenced by article's titles. To test this hypothesis, we investigated if research papers whose findings apply to mice, and not to humans, but which omit this caveat in their titles, generate significantly more news stories with headlines that likewise omit mice, if compared to research papers with titles that do mention mice."

2.Line 23: Should "knowledge" be "acknowledge"
3. Line 106: As for Line 23
4. Line 294: Should "though" be "through"
5. Line 304: SHould "that" be "than" (this does occur elsewhere in a few places and the authors should confirm / check throughout)
6. Line 364: Should "constrain" be "Constraint"
Our answer: Issues numbered 2 to 6 have been taken care of. Thanks. We also carefully reviewed the text again for typos and grammar errors.

7. Lines 437 - 441: (principally line 438) it is not just that the ARRIVE 2.0 "still does not identify the title of a paper as an important place to acknowledge the species, or strain, used in the study" The ARRIVE 2.0 don't even stipulate the title as an element to review!
Our answer: Thanks for raising this important issue. We've made changes to this sentence, as shown below.
The 2020 update to the ARRIVE guidelines [42] does not identify the title of a paper as an important element subjected to review. However, we believe the findings presented here provide grounds for further amendment of the ARRIVE guidelines, together with journal publication policy, to give special attention to the article's title and require the titles of papers describing experimental studies to identify the species and/or tissue sources used in the research.

Answering Quinn Grundy

8.Abstract - I would suggest reordering the first sentences so that the @justinmice sentence comes first as a 'hook' and introduces the paper's main focus - accurate science reporting; then to introduce the content on Alzheimer's disease as essentially the case study you are using to study this (akin to the way you have ordered the Author Summary).
Our answer: Thanks for your suggestion to reorder the abstract. Your suggestion has been accepted, and we believe the abstract flows much better now. Thanks!

9.In the Author Summary, perhaps delete "solid" from "solid data" (let the reader judge this) and reword to avoid suggesting causality/directionality (ie state, "science reporting is associated with media reporting").
Our answer: The editor informed us that there is no author summary anymore and asked us to delete it altogether.

10.In the Introduction, I actually liked the ordering the authors had in the earlier version - a paragraph introducing the main focus (scrutiny of science reporting) and I love the 'hook' with @justinmice. Perhaps you can keep the original first paragraph, then introduce the material on Alzheimer's disease and explain explicitly, why this provides such a good case study for studying the phenomenon of omission of the animal model in titles.
Our answer: We accepted your suggestion and re-arranged the introduction to look more like it was originally presented. We added the phrase below to link the first paragraph to the following ones that discourse about Alzheimer Disease. "This situation, and the use of exaggerated language, is frequently seen in news reporting of health research and Alzheimer Disease (AD) is no exception [4]."

11. It seems the paragraphs beginning line 116 ("This sample) belongs at the beginning of the Results (or could be omitted) and then the section "Our findings" (line 124) in the Discussion or omitted. I would suggest ending the Introduction on line 115.
Our answer: We moved the paragraph that starts with "This sample..." to the beginning of the Results section, as suggested. However, we would like to keep the paragraph that starts with "Our findings..." because we find important to provide in the introduction of the paper a general idea of the results our study has produced.

12. The results needs a first paragraph that explains how you arrived at this sample and the two comparison groups. Again, in meta-research papers, a flow digram is often the most expedient way to communicate this, especially to clearly show how many scientific papers in each group resulted in news pieces (with accompanying 'n's). Could you revise Figure 1 to include all aspects of the sampling process including the sampling frame, how many papers were excluded and reasons why.
Our answer: We have created a new figure (now called figure 1; of a total of 4 figures) that explains the sampling process and how each group of papers was obtained.

13.I had previously raised this, but particularly for descriptive data, including proportions (%) would be meaningful; every proportion should be accompanied by the numerator and denominator so it is really clear to which group or sub-sample you are referring to. It is otherwise very hard to follow.
Our answer: This has been done throughout our text. Thanks for the suggestion.

14.And when providing comparisons, please provide the proportion for each group, e.g "We did find that nondeclarative papers generated significantly more news proportionally (31%) than the papers in the declarative group (X%) (p = 0.012)."
Our answer: This has been done throughout our text. Thanks for the suggestion.

15. In Figure 1, what is the significant of the last row of "News" - what are the two groups?
Our answer: This figure now is called Figure 2. We are sorry if it was not clear. The last row shows the number of news that omitted, or not, mice, in their headlines. This information is not written in the figure itself but is represented by a small drawing of a mouse in the headline. Thus, for the group of declarative papers, 397 news mentioned mice in the headline (note the image of the mouse in the headline) while 465 did not (no image of a mouse in the headline). The same rational apply for the group of news for the nondeclarative papers.
To make these ideas clearer, we added the following sentence in the figure legend: "*Note the drawing of a little mouse in the title and headline of the images representing declarative groups."*

16. For each aspect of the analysis, it would be helpful to have a sub-heading to guide the reader. e.g. to introduce the section beginning "News stories posted online often reproduce the original title of the research paper [15]. . "
Our answer: Thanks for your suggestion. We created the following sub-headings in the Results section.

- **The sampling process**
- **Papers in both groups generate news stories**
- **Nondeclarative papers are more newsworthy**
- **News writers tend to follow article's authors on omitting mice**
- **Journal choice does not explain our findings**
- **Articles' titles omitting mice are more often tweeted**

17. Line 239, "constraint" typo; and "mentioning mice or not" and line 240 "limitations"
Our answer: all fixed, thanks!

18. The first sentence of the discussion 249 is very hard to read; please reword, or just delete. The second sentence makes sense and nicely sums up the main finding.
Our answer: This sentence now reads as follows: " Here we investigated the hypothesis that studies using mice as the main research subject, but which omit this information in their articles' titles, generate significantly more news stories with headlines that likewise omit mice. Our data supports our hypothesis that news writers are influenced by article's titles regarding the omission, or not, of mice in their headlines."

19.This sentence seems contradictory: "Interestingly, the The PLoS guidelines on How to Write a Great Title suggests authors to include the organism used in the research, even though none of the PLoS journals asks authors to add the study's organism to the article's title [18]."

Our answer: We removed this sentence altogether.

20.Line 311- reword, I am not sure that "overclaims" is a word - perhaps "over-interprets"?
Our answer: According to the Merriam-Webster overclaim is a verb, and we wrongly used it as a noun. We fixed the sentence that now reads as follows: " Often, these headlines overclaim the scientific finding and can lead to misperceptions by the general public..." Thank you!

21.You mention "One example is the guidelines created for The Age, The Sydney Morning Herald, Brisbane Times and WAtoday, in Australia." What do these guidelines actually entail?
Our answer: We rewrote this fragment and added more information. We also added the links to the guidelines. This fragment now reads as follows:
"Another example is the guidelines created for The Age, The Sydney Morning Herald, Brisbane Times and WAtoday, in Australia, that among many topics explicitly describes that journalists "... will treat research based on animal studies with caution, preferring to focus on human trials, and making clear that the results may not translate to human trials" [28]. Similarly, Science Media Centre has produced a guideline that describes 10 best practices for reporting science and health stories that includes: "Headlines should not mislead the reader about a story's contents and quotation marks should not be used to dress up overstatement" [29].

22.The material on 340 reporting post-hoc sub-group analysis should be in the Results and the Discussion of these results separated out.
Our answer: Thanks for the suggestion. We did move the paragraph to the Results section and changed the content a little to make it clearer. Now it reads as follows: "In the attempt to explain the association between articles' titles and news' headlines, we raised the hypothesis that press releases in Eurekalert (the main repository for science-related press releases) could influence writers on their news stories. However, we observed that a similar pattern for news stories is also found for Eurekalert press releases, in which declarative papers generated more declarative releases (5 declarative releases out of 22 releases) if compared to nondeclarative papers (1 in 19). We next searched for press releases for any of the 70 articles in the nondeclarative group of papers that generated news stories that had mice in their headlines. We found that only one Eurekalert press release was produced for a single paper among all the 70 nondeclarative research papers. Thus, press releases do not explain the association we found.
We then asked if there was any particularity in these 70 research papers that could have driven writers to write headlines that mentioned mice. Notably, these research papers generated news stories omitting mice in their headlines as well as others acknowledging it, indicating that nothing particular in these papers could be driving writers to choose one way or the other. An initial analysis of the news outlets did not reveal a pattern that could indicate that omitting, or not, mice in the headline was an editorial decision of any kind."

25.The material beginning on line 370 is quite speculative and does not really add much. I would suggest deleting this paragraph.
Our answer: In previous versions we had additional paragraphs in which we presented some hypotheses to explain why authors hide the use of animals in their studies' titles. We removed most of these. This paragraph is the only one left that offers some insight on why some authors omit mice in the title and offers some ideas to those interested in investigating the association presented here further. Thus, we would like to keep this paragraph in our paper.

26.The paragraphs beginning line 397 and 406 should be combined.
Our answer: We accepted your suggestion and now have the paragraphs together.
